# Simultaneous and semi-alternating projection algorithms for solving split equality problems

**DOI:** 10.1186/s13660-017-1595-5

**Published:** 2018-01-04

**Authors:** Qiao-Li Dong, Dan Jiang

**Affiliations:** Tianjin Key Laboratory for Advanced Signal Processing, College of Science, Civil Aviation University of China, Tianjin, 300300 China

**Keywords:** 90C47, 49J35, simultaneous projection algorithm, semi-alternating projection algorithm, maximal monotone operator, split equality problem

## Abstract

In this article, we first introduce two simultaneous projection algorithms for solving the split equality problem by using a new choice of the stepsize, and then propose two semi-alternating projection algorithms. The weak convergence of the proposed algorithms is analyzed under standard conditions. As applications, we extend the results to solve the split feasibility problem. Finally, a numerical example is presented to illustrate the efficiency and advantage of the proposed algorithms.

## Introduction

Let $H_{1}$, $H_{2}$ and $H_{3}$ be real Hilbert spaces, let $C \subseteq H_{1}$ and $Q \subseteq H_{2}$ be two nonempty closed convex sets, and let $A: H_{1}\rightarrow H_{3}$ and $B: H_{2} \rightarrow H_{3}$ be two bounded linear operators.

In this article, we consider the classical split equality problem (SEP), which was first introduced by Moudafi [1]. The SEP can mathematically be formulated as follows:

1 $$ \mbox{Find}\quad x \in C, y \in Q \quad \mbox{such that} \quad Ax=By . $$

Throughout this paper, assume that SEP (1) is consistent and denote by

$$\Gamma=\{ x\in C,y\in Q: Ax=By\} $$

the solution of SEP (1). Then Γ is closed, convex and nonempty.

The split equality problem (1) is actually an optimization problem with weak coupling in the constraint (see [1] for details) and its interest covers many situations, for instance, in domain decomposition for PDEs, game theory and intensity-modulated radiation therapy (IMRT). In domain decomposition for PDEs, this equals to the variational form of a PDE in a domain that can be decomposed into two non-overlapping subdomains with a common interface (see, e.g., [2]). In decision sciences, this allows to consider agents who interplay only via some components of their decision variables (see, e.g., [3]). In IMRT, this amounts to envisaging a weak coupling between the vector of doses absorbed in all voxels and that of the radiation intensity (see [4] for further details). Attouch [5] pointed out more applications of the SEP in optimal control theory, surface energy and potential games, whose variational form can be seen as a SEP.

Next we present an example, in which a separable optimization problem can be rewritten as a split equality problem.

### Example 1.1

Consider the separable optimization problem

2 $$ \begin{aligned} &\mbox{minimize } f(x)+g(y) \\ &\quad \mbox{subject to } Ax=By, \end{aligned} $$

with $x \in R^{N}$ and $y \in R^{M}$, where $A\in R^{J\times N}$ and $B\in R^{J\times M}$. Assume that *f* and *g* are convex and the solution set of problem (2) is nonempty.

Set $C=\operatorname*{argmin}\{f(x) \mid x\in R^{N} \}$ and $Q=\operatorname*{argmin}\{g(y) \mid y\in R^{M} \}$. Then the optimization problem (2) equals to the following split equality problem:

3 $$ \mbox{Find}\quad x \in C, y \in Q \quad \mbox{such that} \quad Ax=By. $$

A great deal of literature on algorithms for solving SEP has been published, most of which are projection methods [1–3, 6–11]. Based on the classical projection gradient algorithm, Byrne and Moudafi [12] introduced the following algorithm, which is also called the simultaneous iterative method [13]:

4 $$ \textstyle\begin{cases} x^{k+1}=P_{C}(x^{k}-\beta_{k} A^{*}( A x^{k}-B y^{k})), \\ y^{k+1}=P_{Q}(y^{k}+\beta_{k} B^{*}(A x^{k}-B y^{k})), \end{cases} $$

where $\beta_{k}\in(\varepsilon,{2}/({\lambda_{A}+\lambda _{B}})-\varepsilon)$, $\lambda_{A}$ and $\lambda_{B}$ are the operator (matrix) norms $\|A\|$ and $\|B\|$ (or the largest eigenvalues of $A^{*}A$ and $B^{*}B$), respectively. To determine stepsize $\beta_{k}$, one needs first to calculate (or estimate) the operator norms $\|A\|$ and $\|B\|$. In general, it is difficult or even impossible. On the other hand, even if we know the norm of *A* and *B*, the algorithm (4) method with fixed stepsize may be slow.

In order to deal with this, the authors [9] introduced a self-adaptive projection algorithm, in which the stepsize is computed by using an Armijo search.

Define the function $F:H_{1}\times H_{2} \rightarrow H_{1}$ by

$$F(x,y)=A^{*}(Ax-By), $$

and the function $G:H_{1}\times H_{2} \rightarrow H_{2}$ by

$$G(x,y)=B^{*}(By-Ax). $$

The self-adaptive projection algorithm in [9] is defined as follows.

### Algorithm 1.1

Given constants $\sigma_{0} > 0$, $\alpha\in(0, 1)$, $\theta\in(0, 1)$ and $\rho\in(0,1)$. Let $x^{0}\in H_{1}$ and $y^{0}\in H_{2}$ be taken arbitrarily. For $k=0,1,2,\ldots$ , compute

5 $$ \textstyle\begin{cases} u^{k}=P_{C}(x^{k}-\beta_{k}F(x^{k},y^{k})), \\ v^{k}=P_{Q}(y^{k}-\beta_{k}G(x^{k},y^{k})), \end{cases} $$

where $\beta_{k}$ is chosen to be the largest $\beta\in\{\sigma_{k},\sigma _{k}\alpha,\sigma_{k}\alpha^{2},\ldots\}$ satisfying

6 $$\begin{aligned}& {\beta^{2}}\bigl( \bigl\Vert F\bigl(x^{k},y^{k} \bigr)-F\bigl(u^{k},v^{k}\bigr) \bigr\Vert ^{2}+ \bigl\Vert G\bigl(x^{k},y^{k}\bigr)-G\bigl(u^{k},v^{k} \bigr) \bigr\Vert ^{2}\bigr) \\& \quad \leq \theta^{2}\bigl({ \bigl\Vert x^{k}-u^{k} \bigr\Vert ^{2}+ \bigl\Vert y^{k}-v^{k} \bigr\Vert ^{2}}\bigr). \end{aligned}$$

Compute

7 $$ \textstyle\begin{cases} x^{k+1}=P_{C}(x^{k}-\beta_{k}F(u^{k},v^{k})), \\ y^{k+1}=P_{Q}(y^{k}-\beta_{k}G(u^{k},v^{k})). \end{cases} $$

If

8 $$\begin{aligned}& {\beta_{k}^{2}}\bigl( \bigl\Vert F\bigl(x^{k},y^{k} \bigr)-F\bigl(x^{k+1},y^{k+1}\bigr) \bigr\Vert ^{2}+ \bigl\Vert G\bigl(x^{k},y^{k}\bigr)-G\bigl(x^{k+1},y^{k+1} \bigr) \bigr\Vert ^{2}\bigr) \\& \quad \leq\rho^{2}\bigl({ \bigl\Vert x^{k}-x^{k+1} \bigr\Vert ^{2}+ \bigl\Vert y^{k}-y^{k+1} \bigr\Vert ^{2}}\bigr), \end{aligned}$$

then set $\sigma_{k}=\sigma_{0}$; otherwise, set $\sigma_{k}=\beta_{k}$.

In fact, Algorithm 1.1 can be seen as an extension of the classical extragradient method first proposed by Korpelevich [14]. Notice that, in Algorithm 1.1, the stepsize of the prediction (5) and that of the correction (7) are equal. Thus these two steps seem to be ‘symmetric’.

Recently, Chuang and Du [15] proposed the following projection algorithm (which is called the hybrid projected Landweber algorithm).

### Algorithm 1.2

Given constants $\sigma>0, \alpha\in(0,1)$ and $\theta\in(0,1)$, let $x^{0} \in H_{1}$ and $y^{0}\in H_{2}$ be taken arbitrarily. For $k = 0,1,2,\ldots $ , compute

$$\textstyle\begin{cases} u^{k}=P_{C}(x^{k}-\beta_{k} F(x^{k},y^{k})), \\ v^{k}=P_{Q}(y^{k}-\beta_{k} G(x^{k},y^{k})), \end{cases} $$

where $\beta_{k}$ is chosen via (6) and (8). Compute next iterates $x^{k+1}$ and $y^{k+1}$ by

9 $$ \textstyle\begin{cases} x^{k+1}=P_{C}(x^{k}-\rho_{k} c_{k}), \\ y^{k+1}=P_{Q}(y^{k}-\rho_{k} d_{k}), \end{cases} $$

where

$$\textstyle\begin{cases} c_{k}:=(x^{k}-u^{k})-\beta_{k}(F(x^{k},y^{k})-F(u^{k},v^{k})); \\ d_{k}:=(y^{k}-v^{k})-\beta_{k}(G(x^{k},y^{k})-G(u^{k},v^{k})), \end{cases} $$

and

10 $$ \rho_{k}:=\frac{\langle x^{k}-u^{k}, c_{k}\rangle+\langle y^{k}-v^{k}, d_{k}\rangle }{\|c_{k}\|^{2}+\|d_{k}\|^{2}}. $$

Note that Algorithm 1.2 with $\rho_{k}\equiv1$ in (9) can be seen as a special case of Tseng’s method [8, 16]. The projections in the second step of Tseng’s method are made onto two nonempty closed convex sets $X\subseteq H_{1}$ and $Y\subseteq H_{2}$, other than *C* and *Q*. *X* and *Y* can be any sets such that the intersections of *X* and *C* (and *Y* and *Q*) are nonempty, and they may be taken to have simple structures so that the projections onto them are easy to calculate.

Chuang and Du [15] proved the convergence of Algorithm 1.2 and also presented the convergence property of Algorithm 1.2 as follows:

11 $$\begin{aligned}& \bigl\Vert x^{k+1}-x^{*} \bigr\Vert ^{2}+ \bigl\Vert y^{k+1}-y^{*} \bigr\Vert ^{2} \\& \quad \leq \bigl\Vert x^{k}-x^{*} \bigr\Vert ^{2}+ \bigl\Vert y^{k}-y^{*} \bigr\Vert ^{2}-\rho_{k}^{2} \bigl( \Vert c_{k} \Vert ^{2}+ \Vert d_{k} \Vert ^{2}\bigr), \end{aligned}$$

where $(x^{*},y^{*})\in\Gamma$.

The stepsize $\beta_{k}$ in Algorithms 1.1 and 1.2 is obtained through the Armijo search (6). In general, the computational cost of a self-adaptive algorithm is large, since one may need to calculate (5) several times to get the stepsize $\beta_{k}$.

To overcome this difficulty, the authors [17] introduced a projection algorithm for which the stepsize does not depend on the operator norms $\|A\|$ and $\|B\|$, and one can directly compute the stepsize instead of using the Armijo search.

### Algorithm 1.3

Choose initial guesses $x^{0}\in H_{1}, y^{0}\in H_{2}$ arbitrarily. Assume that the *k*th iterate $x^{k} \in C$, $y^{k}\in Q$ has been constructed and $Ax^{k}-By^{k}\neq0$; then we calculate the $(k + 1)$th iterate $(x^{k+1},y^{k+1})$ via the formula

12 $$ \textstyle\begin{cases} x^{k+1}=P_{C}(x^{k}-\beta_{k} A^{*}(Ax^{k}-By^{k})), \\ y^{k+1}=P_{Q}(y^{k}+\beta_{k} B^{*}(Ax^{k}-By^{k})), \end{cases} $$

where the stepsize $\beta_{k}$ is chosen in such a way that

13 $$ \beta_{k}=\sigma_{k}\min \biggl\{ \frac{ \Vert Ax^{k}-By^{k} \Vert ^{2}}{ \Vert A^{*}(Ax^{k}-By^{k}) \Vert ^{2}}, \frac{ \Vert Ax^{k}-By^{k} \Vert ^{2}}{ \Vert B^{*}(Ax^{k}-By^{k}) \Vert ^{2}} \biggr\} , $$

where $0<\sigma_{k}<1$. If $Ax^{k}-By^{k}= 0$, then $(x^{k+1},y^{k+1})=(x^{k},y^{k})$ is a solution of SEP (1) and the iterative process stops; otherwise, we set $k := k + 1$ and go onto (12) to evaluate the next iterate $(x^{k+2},y^{k+2})$.

Note that the choice in (13) of the stepsize $\beta_{k}$ is independent of the norms $\|A\|$ and $\|B\|$.

Polyak [18, 19] first introduced the inertial extrapolation algorithms, which were widely studied as an acceleration process. The authors [20] made an inertial modification for Algorithm 1.3 and introduced the following inertial projection methods for SEP.

### Algorithm 1.4

Choose initial guesses $x^{0},x^{1}\in H_{1}$, $y^{0},y^{1}\in H_{2}$ arbitrarily. Compute

14 $$ \textstyle\begin{cases} (\bar{x}^{k},\bar{y}^{k})=(x^{k},y^{k})+\alpha_{k}(x^{k}-x^{k-1},y^{k}-y^{k-1}), \\ x^{k+1}=P_{C}(\bar{x}^{k}-\beta_{k} A^{*}( A\bar{x}^{k}-B\bar{y}^{k})), \\ y^{k+1}=P_{Q}(\bar{y}^{k}+\beta_{k}B^{*}(A\bar{x}^{k}-B\bar{y}^{k})), \end{cases} $$

where $\alpha_{k}\in(0,1)$ and the stepsize $\gamma_{k}$ is chosen in the same way as (13).

They showed the weak convergence of Algorithm 1.4 under some conditions on the inertial parameter $\alpha_{k}$.

In fact, Algorithm 1.4 can be seen as a FISTA (fast iterative shrinkage-thresholding algorithm) introduced by Beck and Teboulle [21] to solve the linear inverse problems, if we take the inertial parameter $\alpha_{k}=\frac{t_{k}-1}{t_{k+1}}$, where $t_{1}=1$ and $t_{k+1}=\frac{1+\sqrt{1+4t_{k}^{2}}}{2}$, $k\geq1 $, and choose a constant stepsize $\beta_{k}$ or choose $\beta_{k}$ via a backtracking stepsize rule. A shortcoming of the method of Beck and Teboulle is that they could not prove the convergence of the iterative sequence $(x^{k},y^{k})$. Chambolle and Dossal [22] improved the choice of the inertial parameter, took $\alpha_{k}=\frac{k-1}{k+a}$, where $a>2$, and presented the convergence of the iterative sequence $(x^{k},y^{k})$.

In this paper, inspired by the work in [17, 23, 24], we introduce two simultaneous projection algorithms by improving the stepsizes $\beta_{k}$ and $\rho_{k}$ of the second step (7) and (9) in Algorithms 1.1 and 1.2, respectively. We also present two alternating projection algorithms, in which we take an alternating technique in the first step.

The structure of the paper is as follows. In the next section, we present some concepts and lemmas which will be used in the main results. In Section 3, two classes of projection algorithms are provided and their weak convergence is analyzed. In Section 4, we extend the results to the split feasibility problem. In the final section, some numerical results are provided, which show the advantages of the proposed algorithms.

## Preliminaries

Let *H* be a real Hilbert space with the inner product $\langle\cdot ,\cdot\rangle$ and the induced norm $\Vert\cdot\Vert$, and let *D* be a nonempty, closed and convex subset of *H*. We write $x^{k}\rightharpoonup x$ to indicate that the sequence $\{ x^{k} \} _{k=0}^{\infty}$ converges weakly to *x* and $x^{k}\rightarrow x$ to indicate that the sequence $\{ x^{k} \} _{k=0}^{\infty}$ converges strongly to *x*. Given a sequence $\{ x^{k} \} _{k=0}^{\infty}$, denote by $\omega_{w}(x^{k})$ its weak *ω*-limit set, that is, any $x\in\omega_{w}(x^{k})$ such that there exists a subsequence $\{ x^{k_{j}} \} _{j=0}^{\infty}$ of $\{ x^{k} \} _{k=0}^{\infty}$ which converges weakly to *x*.

In this paper, an important tool of our work is the projection. Let *H* be a real Hilbert space and *C* be a closed convex subset of *H*. Recall that the projection from *H* onto *C*, denoted by $P_{C}$, is defined in such a way that, for each $x\in H$, $P_{C}(x)$ is the unique point in *C* such that

$$\bigl\Vert x-P_{C}(x) \bigr\Vert =\min\bigl\{ \Vert x-z \Vert :z\in C\bigr\} . $$

The following two lemmas are useful characterizations of projections.

### Lemma 2.1

([25])

*Given* $x\in H$ *and* $z\in C$. *Then* $z=P_{C}(x)$ *if and only if*

$$\langle x-z,y-z\rangle\leq0,\quad \forall y\in C. $$

### Lemma 2.2

([25, 26])

*For any* $x,y\in H$ *and* $z\in C$, *it holds*

(i) $\|P_{C}(x)-P_{C}(y)\|\leq\|x-y\|$;
(ii) $\|P_{C}(x)-z\|^{2}\leq\|x-z\|^{2}-\|P_{C}(x)-x\|^{2}$.

### Definition 2.1

The normal cone of *C* at $v\in C$, denoted by $N_{C} ( v )$, is defined as

$$N_{C} ( v ) :=\bigl\{ d\in H\mid \langle d,y-v \rangle\leq0\text{ for all }y\in C\bigr\} . $$

### Definition 2.2

Let $A:H\rightrightarrows2^{H}$ be a point-to-set operator defined on a real Hilbert space *H*. The operator *A* is called a maximal monotone operator if *A* is monotone, i.e.,

$$\langle u-v,x-y \rangle\geq0\quad \text{for all }u\in A(x)\text{ and }v\in A(y), $$

and the graph $G(A)$ of *A*,

$$G(A):= \bigl\{ ( x,u ) \in H\times H\mid u\in A(x) \bigr\} , $$

is not properly contained in the graph of any other monotone operator.

It is clear [27, Theorem 3] that a monotone mapping *A* is maximal if and only if, for any $( x,u ) \in H\times H$, if $\langle u-v,x-y \rangle\geq0$ for all $( v,y ) \in G(A)$, then it follows that $u\in A(x)$.

### Lemma 2.3

([26])

*Let* *D* *be a nonempty*, *closed and convex subset of a Hilbert space* *H*. *Let* $(x^{k})$ *be a bounded sequence which satisfies the following properties*:

(i) *every limit point of* $\{x^{k}\}_{k=0}^{\infty}$ *lies in* *D*;
(ii) $\lim_{n\rightarrow\infty}\Vert x^{k}-x\Vert$ *exists for every* $x\in D$.

*Then* $\{x^{k}\}$ *converges weakly to a point in* *D*.

## Main results

In this section, we present two classes of projection algorithms and establish their weak convergence under standard conditions.

### Simultaneous projection algorithms

Let $S= C\times Q\in H := H_{1}\times H_{2}$. Define $K =[A,-B] : H_{1} \times H_{2} \rightarrow H_{1} \times H_{2}$, and let $K^{*}$ be the adjoint operator of *K*, then the original problem (1) can be modified as

15 $$ \mbox{Find} \quad z=(x,y)\in S \quad \mbox{such that} \quad Kw=0. $$

Note that if the solution set of (15) is nonempty, it equals the following minimization problem:

16 $$ \min_{z\in S}\frac{1}{2}\|Kz \|^{2}, $$

which is a standard (and a simple) problem from the convex optimization point of view. There are many methods for solving the minimization problem (16) such as the classical projection gradient method. Algorithm (4) (also Algorithm 1.3) is the exact projection gradient method when applied to (16).

Inspired by Cai [24] and Dong et al. [17], we propose two new simultaneous projection algorithms by improving the stepsizes in the second step of Algorithms 1.1 and 1.2.

#### Algorithm 3.1

Given constants $\sigma>0$, $\alpha\in(0,1)$, $\theta\in(0,1)$ and $\rho\in(0,1)$, let $z_{0}=(x^{0},y^{0}) \in H=H_{1}\times H_{2}$ be taken arbitrarily.

For $k = 0,1,2,\ldots $ , compute

17 $$ w^{k}=P_{S}\bigl(z^{k}- \beta_{k} K^{*}K\bigl(z^{k}\bigr)\bigr), $$

where $\beta_{k}$ is chosen to be the largest $\beta\in\{\sigma_{k},\sigma _{k}\alpha,\sigma_{k}\alpha^{2},\ldots \}$ satisfying

18 $$ \beta \bigl\Vert K^{*}K\bigl(z^{k}\bigr)-K^{*}K \bigl(w^{k}\bigr) \bigr\Vert \leq\theta \bigl\Vert z^{k}-w^{k} \bigr\Vert . $$

Compute next iterates $z^{k+1}$ by

19 $$ z^{k+1}_{\mathrm{I}}=z^{k}-\gamma \rho_{k} d\bigl(z^{k},w^{k}\bigr), $$

or

20 $$ z^{k+1}_{\mathrm{II}}=P_{S} \bigl(z^{k}-\gamma\beta_{k}\rho_{k} K^{*}K \bigl(w^{k}\bigr)\bigr), $$

where $\gamma\in[0,2)$,

21 $$ d\bigl(z^{k},w^{k}\bigr):= \bigl(z^{k}-w^{k}\bigr)-\beta_{k}\bigl(K^{*}K \bigl(z^{k}\bigr)-K^{*}K\bigl(w^{k}\bigr)\bigr), $$

and

22 $$ \rho_{k}:=\frac{\langle z^{k}-w^{k}, d(z^{k},w^{k})\rangle+\beta_{k} \Vert K(w^{k}) \Vert ^{2}}{ \Vert d(z^{k},w^{k}) \Vert ^{2}}. $$

If

23 $$ \beta_{k} \bigl\Vert K^{*}K\bigl(z^{k} \bigr)-K^{*}K\bigl(z^{k+1}\bigr) \bigr\Vert \leq\rho \bigl\Vert z^{k}-z^{k+1} \bigr\Vert , $$

then set $\sigma_{k}=\sigma_{0}$; otherwise, set $\sigma_{k}=\beta_{k}$.

#### Remark 3.1

Let $z=(x,y)$. Then we have (see Section 4.4.1 in [28])

$$P_{S}(z)=(P_{C}x,P_{Q}y). $$

It is easy to see

$$K^{*}Kz= \left ( \textstyle\begin{array}{@{}c@{\quad}c@{}} A^{*}A&-A^{*}B\\ -B^{*}A&B^{*}B \end{array}\displaystyle \right ) \left ( \textstyle\begin{array}{@{}c@{}} x\\ y \end{array}\displaystyle \right ) = \left ( \textstyle\begin{array}{@{}c@{}} A^{*}(Ax-By)\\ B^{*}(By-Ax) \end{array}\displaystyle \right ). $$

Define the function $F: H_{1} \times H_{2} \rightarrow H_{1}$ by

24 $$ F(x,y)=A^{*}(Ax-By), $$

and the function $G: H_{1} \times H_{2} \rightarrow H_{2}$ by

25 $$ G(x,y)=B^{*}(By-Ax). $$

By setting $z^{k}=(x^{k},y^{k})$ and $w^{k}=(u^{k},v^{k})$, Algorithm 3.1 can be rewritten as follows:

For $k = 0,1,2,\ldots $ , compute

26 $$ \textstyle\begin{cases} u^{k}=P_{C}(x^{k}-\beta_{k} F(x^{k},y^{k})),\\ v^{k}=P_{Q}(y^{k}-\beta_{k} G(x^{k},y^{k})), \end{cases} $$

where $\beta_{k}$ is chosen to be the largest $\beta\in\{\sigma_{k},\sigma _{k}\alpha,\sigma_{k}\alpha^{2},\ldots \}$ satisfying

27 $$\begin{aligned}& \beta^{2}\bigl( \bigl\Vert F\bigl(x^{k},y^{k} \bigr)-F\bigl(u^{k},v^{k}\bigr) \bigr\Vert ^{2}+ \bigl\Vert G\bigl(x^{k},y^{k}\bigr)-G\bigl(u^{k},v^{k} \bigr) \bigr\Vert ^{2}\bigr) \\& \quad \leq \theta^{2}\bigl( \bigl\Vert x^{k}-u^{k} \bigr\Vert ^{2}+ \bigl\Vert y^{k}-v^{k} \bigr\Vert ^{2}\bigr). \end{aligned}$$

Compute next iterates $x^{k+1}$ and $y^{k+1}$ by

28 $$ \textstyle\begin{cases} x^{k+1}_{\mathrm{I}}=x^{k}-\gamma\rho_{k} c_{k}, \\ y^{k+1}_{\mathrm{I}}=y^{k}-\gamma\rho_{k} d_{k}, \end{cases} $$

or

29 $$ \textstyle\begin{cases} x^{k+1}_{\mathrm{II}}=P_{C}(x^{k}-\gamma\beta _{k}\rho_{k} F(u^{k},v^{k})), \\ y^{k+1}_{\mathrm{II}}=P_{Q}(y^{k}-\gamma\beta _{k}\rho_{k} G(u^{k},v^{k})), \end{cases} $$

where $\gamma\in[0,2)$,

30 $$ \textstyle\begin{cases} c_{k}:=(x^{k}-u^{k})-\beta_{k}(F(x^{k},y^{k})-F(u^{k},v^{k})); \\ d_{k}:=(y^{k}-v^{k})-\beta_{k}(G(x^{k},y^{k})-G(u^{k},v^{k})), \end{cases} $$

and

31 $$ \begin{aligned}\rho_{k}:=\frac{\langle x^{k}-u^{k}, c_{k}\rangle+\langle y^{k}-v^{k}, d_{k}\rangle +\beta_{k}\| Au^{k}-Bv^{k}\|^{2}}{\|c_{k}\|^{2}+\|d_{k}\|^{2}}.\end{aligned}$$

If

32 $$\begin{aligned}& {\beta_{k}^{2}}\bigl( \bigl\Vert F\bigl(x^{k},y^{k} \bigr)-F\bigl(x^{k+1},y^{k+1}\bigr) \bigr\Vert ^{2}+ \bigl\Vert G\bigl(x^{k},y^{k}\bigr)-G\bigl(x^{k+1},y^{k+1} \bigr) \bigr\Vert ^{2}\bigr) \\& \quad \leq\rho^{2}\bigl({ \bigl\Vert x^{k}-x^{k+1} \bigr\Vert ^{2}+ \bigl\Vert y^{k}-y^{k+1} \bigr\Vert ^{2}}\bigr), \end{aligned}$$

then set $\sigma_{k}=\sigma_{0}$; otherwise, set $\sigma_{k}=\beta_{k}$.

For convenience, we call the projection algorithms which use update forms (19) (or (28)) and (20) (or (29)) Algorithm 3.1(I) and Algorithm 3.1(II), respectively.

#### Remark 3.2

For Algorithm 3.1, we can get the following conclusions:

(i) The only difference between Algorithm 3.1(II) and Algorithm 1.1 is that they use different stepsizes in the definitions of $x^{k+1}$ and $y^{k+1}$.
(ii) There are two differences between Algorithm 3.1(I) and Algorithm 1.2. Firstly, the stepsize $\rho_{k}$ in (10) of Algorithm 3.1(I) is larger than that in (36) of Algorithm 1.2. Secondly, there are no projections on the second step (19).

#### Remark 3.3

By the definitions of $d_{k}$ in (21), the projection equation (17) can be written as

$$w^{k}=P_{S}\bigl(w^{k}-\bigl(\beta_{k} K^{*}K\bigl(w^{k}\bigr)-d\bigl(z^{k},w^{k}\bigr) \bigr)\bigr). $$

So, from Lemma 2.1 we have

33 $$ \bigl\langle z-w^{k},\beta_{k} K^{*}K \bigl(w^{k}\bigr)-d\bigl(z^{k},w^{k}\bigr)\bigr\rangle \geq0,\quad \forall z \in S. $$

#### Lemma 3.1

*The search rule* (18) *is well defined*. *Besides* $\underline{\beta }\leq\beta_{k}\leq\sigma$, *where*

34 $$ \underline{\beta}=\min\biggl\{ \sigma, \frac{\alpha\theta}{\|K\|^{2}}\biggr\} . $$

#### Proof

Obviously, $\beta_{k}\leq\sigma_{k}\leq\sigma_{0}$. In the latter case, we know that $\beta_{k}/\alpha$ must violate inequality (18), that is,

35 $$ \bigl\Vert K^{*}K\bigl(z^{k}\bigr)-K^{*}K \bigl(w^{k}\bigr) \bigr\Vert \geq\theta\frac{ \Vert z^{k}-w^{k} \Vert }{\beta_{k}/\alpha}. $$

On the other hand, we have

36 $$ \bigl\Vert K^{*}K\bigl(z^{k}\bigr)-K^{*}K \bigl(w^{k}\bigr) \bigr\Vert \leq \Vert K \Vert ^{2} \bigl\Vert z^{k}-w^{k} \bigr\Vert . $$

Consequently, we get (34). □

#### Lemma 3.2

*Let* $(z^{k})$ *and* $(w^{k})$ *be generated by Algorithm* 3.1, *and let* $d_{k}$ *and* $\rho_{k}$ *be given by* (21) *and* (36), *respectively*. *Then we have*

37 $$ \rho_{k}\geq\frac{1-\theta}{1+\theta^{2}}. $$

#### Proof

By the Cauchy-Schwarz inequality, we have

38 $$\begin{aligned} & \bigl\langle z^{k}-w^{k}, d\bigl(z^{k},w^{k} \bigr)\bigr\rangle \\ &\quad = \bigl\Vert z^{k}-w^{k} \bigr\Vert ^{2}-\beta_{k}\bigl\langle z^{k}-w^{k},K^{*}K \bigl(z^{k}\bigr)-K^{*}K\bigl(w^{k}\bigr)\bigr\rangle \\ &\quad \geq \bigl\Vert z^{k}-w^{k} \bigr\Vert ^{2}-\beta_{k} \bigl\Vert z^{k}-w^{k} \bigr\Vert \bigl\Vert K^{*}K\bigl(z^{k}\bigr)-K^{*}K \bigl(w^{k}\bigr) \bigr\Vert \\ &\quad \geq \bigl\Vert z^{k}-w^{k} \bigr\Vert ^{2}-\theta \bigl\Vert z^{k}-w^{k} \bigr\Vert ^{2} \\ &\quad =(1-\theta) \bigl\Vert z^{k}-w^{k} \bigr\Vert ^{2}. \end{aligned}$$

By using $\langle z^{k}-w^{k},K^{*}K(z^{k})-K^{*}K(w^{k})\rangle=\langle K(z^{k})-K(w^{k}),K(z^{k})-K(w^{k})\rangle=\|K(z^{k})-K(w^{k})\|^{2}$, we have

$$\begin{aligned} \bigl\Vert d\bigl(z^{k},w^{k}\bigr) \bigr\Vert ^{2} =& \bigl\Vert z^{k}-w^{k} \bigr\Vert ^{2}+\beta_{k}^{2} \bigl\Vert K^{*}K \bigl(z^{k}\bigr)-K^{*}K\bigl(w^{k}\bigr) \bigr\Vert ^{2} \\ &{} -2\beta_{k}\bigl\langle z^{k}-w^{k},K^{*}K \bigl(z^{k}\bigr)-K^{*}K\bigl(w^{k}\bigr)\bigr\rangle \\ \leq& \bigl\Vert z^{k}-w^{k} \bigr\Vert ^{2}+ \theta^{2} \bigl\Vert z^{k}-w^{k} \bigr\Vert ^{2}-2\beta_{k} \bigl\Vert K\bigl(z^{k}\bigr)-K \bigl(w^{k}\bigr) \bigr\Vert ^{2} \\ \leq&\bigl(1+\theta^{2}\bigr) \bigl\Vert z^{k}-w^{k} \bigr\Vert ^{2}. \end{aligned}$$

So, we get (37). □

#### Lemma 3.3

*Let* $(z^{k})$ *and* $(w^{k})$ *be generated by Algorithm* 3.1, *and let* $d_{k}$ *be given by* (21). *Then*, *for all* $(z^{*})\in\Gamma$, *we have*

39 $$ \bigl\langle z^{k}-z^{*}, d\bigl(z^{k},w^{k} \bigr)\bigr\rangle \geq\rho_{k} \bigl\Vert d\bigl(z^{k},w^{k} \bigr) \bigr\Vert ^{2}. $$

#### Proof

Take arbitrarily $z^{*}\in\Gamma$, that is, $z^{*}\in S$, and $K(z^{*})=0$. By setting $z=z^{*}$ in (33), we get

$$\bigl\langle z^{*}-w^{k},\beta_{k}K^{*} K\bigl(w^{k} \bigr)-d\bigl(z^{k},w^{k}\bigr)\bigr\rangle \geq0, $$

which implies that

$$\bigl\langle w^{k}-z^{*},d\bigl(z^{k},w^{k}\bigr) \bigr\rangle \geq\beta_{k} \bigl\langle w^{k}-z^{*},K^{*}K \bigl(w^{k}\bigr)\bigr\rangle . $$

It is easy to show that

40 $$ \bigl\langle w^{k}-z^{*},K^{*}K\bigl(w^{k}\bigr) \bigr\rangle =\bigl\langle K\bigl(w^{k}-z^{*}\bigr),K\bigl(w^{k} \bigr)\bigr\rangle = \bigl\Vert K\bigl(w^{k}\bigr) \bigr\Vert ^{2}. $$

So we have

$$\begin{aligned} \bigl\langle z^{k}-z^{*},d\bigl(z^{k},w^{k}\bigr) \bigr\rangle &=\bigl\langle z^{k}-w^{k},d\bigl(z^{k},w^{k} \bigr)\bigr\rangle +\bigl\langle w^{k}-z^{*},d\bigl(z^{k},w^{k} \bigr)\bigr\rangle \\ &\geq\bigl\langle z^{k}-w^{k},d\bigl(z^{k},w^{k} \bigr)\bigr\rangle +\beta_{k} \bigl\Vert K\bigl(w^{k}\bigr) \bigr\Vert ^{2} \\ &=\rho_{k} \bigl\Vert d\bigl(z^{k},w^{k}\bigr) \bigr\Vert ^{2}, \end{aligned}$$

which implies (39). □

#### Theorem 3.1

*Let* $(z^{k})$ *be generated by Algorithm* 3.1(I). *If* Γ *is nonempty*, *then we have*

41 $$ \bigl\Vert z^{k+1}_{\mathrm{I}}-z^{*} \bigr\Vert ^{2}\leq \bigl\Vert z^{k}-z^{*} \bigr\Vert ^{2}- \gamma(2-\gamma)\rho_{k}^{2} \bigl\Vert d \bigl(z^{k},w^{k}\bigr) \bigr\Vert ^{2},\quad \forall z^{*}\in\Gamma $$

*and* $(z^{k})$ *converges weakly to a solution of SEP* (1).

#### Proof

Let $z^{*} \in\Gamma$, that is, $z^{*}\in S$, and $K(z^{*})=0$. Then, from (39), we have

42 $$\begin{aligned} \begin{aligned}[b] \bigl\Vert z^{k+1}_{\mathrm{I}}-z^{*} \bigr\Vert ^{2} &= \bigl\Vert z^{k}-z^{*} \bigr\Vert ^{2}+\gamma^{2} \rho_{k}^{2} \bigl\Vert d\bigl(z^{k},w^{k} \bigr) \bigr\Vert ^{2}-2\gamma\rho_{k}\bigl\langle z^{k}-z^{*},d\bigl(z^{k},w^{k}\bigr)\bigr\rangle \\ &\leq \bigl\Vert z^{k}-z^{*} \bigr\Vert ^{2}+ \gamma^{2}\rho_{k}^{2} \bigl\Vert d \bigl(z^{k},w^{k}\bigr) \bigr\Vert ^{2}-2\gamma \rho_{k}^{2} \bigl\Vert d\bigl(z^{k},w^{k} \bigr) \bigr\Vert ^{2} \\ &= \bigl\Vert z^{k}-z^{*} \bigr\Vert ^{2}-\gamma(2-\gamma) \rho_{k}^{2} \bigl\Vert d\bigl(z^{k},w^{k} \bigr) \bigr\Vert ^{2}, \end{aligned} \end{aligned}$$

which yields (41). Since $\gamma\in(0,2)$, (42) implies that the sequence $(\| z^{k}-z^{*}\|^{2})$ is decreasing and thus converges. Moreover, $(z^{k})$ is bounded. This implies that

43 $$ \lim_{k\rightarrow\infty}\rho_{k}^{2} \bigl\Vert d\bigl(z^{k},w^{k}\bigr) \bigr\Vert ^{2}=0. $$

From the definition of $\rho_{k}$, Lemmas 3.1 and 3.2, we have

$$\begin{aligned} \rho_{k}^{2} \bigl\Vert d\bigl(z^{k},w^{k} \bigr) \bigr\Vert ^{2} &=\rho_{k}\bigl(\bigl\langle z^{k}-w^{k}, d\bigl(z^{k},w^{k}\bigr) \bigr\rangle +\beta_{k} \bigl\Vert K\bigl(w^{k}\bigr) \bigr\Vert ^{2}\bigr) \\ &\geq\rho_{k} \bigl[(1-\theta) \bigl\Vert z^{k}-w^{k} \bigr\Vert ^{2}+\beta_{k} \bigl\Vert K \bigl(w^{k}\bigr) \bigr\Vert ^{2} \bigr] \\ &\geq\frac{(1-\theta)^{2}}{1+\theta^{2}} \bigl\Vert z^{k}-w^{k} \bigr\Vert ^{2}+\frac{1-\theta }{1+\theta^{2}}\underline{\beta} \bigl\Vert K \bigl(w^{k}\bigr) \bigr\Vert ^{2}, \end{aligned}$$

which implies

$$\bigl\Vert z^{k}-w^{k} \bigr\Vert ^{2}\leq \frac{1+\theta^{2}}{(1-\theta)^{2}}\rho_{k}^{2} \bigl\Vert d \bigl(z^{k},w^{k}\bigr) \bigr\Vert ^{2} $$

and

$$\bigl\Vert K\bigl(w^{k}\bigr) \bigr\Vert ^{2}\leq \frac{1+\theta^{2}}{(1-\theta)\underline{\beta}}\rho_{k}^{2} \bigl\Vert d \bigl(z^{k},w^{k}\bigr) \bigr\Vert ^{2}. $$

Using (43), we get

44 $$ \lim_{k\rightarrow\infty} \bigl\Vert z^{k}-w^{k} \bigr\Vert =0 $$

and

$$\lim_{k\rightarrow\infty} \bigl\Vert K\bigl(w^{k}\bigr) \bigr\Vert =0. $$

By the boundedness of *K*, we get

45 $$ \lim_{k\rightarrow\infty} \bigl\Vert K\bigl(z^{k} \bigr) \bigr\Vert =0. $$

Let $\hat{z}\in\omega_{w}(z^{k})$, then there exists a subsequence $(z^{k_{i}})$ of $(z^{k})$ which converges weakly to *ẑ*. From (44), it follows that the subsequence $(w^{k_{i}})$ also converges weakly to *ẑ*. We will show that *ẑ* is a solution of SEP (1). The weak convergence of $(K(z^{k_{i}}))$ to $K(\hat{z})$ and lower semicontinuity of the squared norm imply that

$$\bigl\Vert K(\hat{z}) \bigr\Vert \leq\liminf_{i\rightarrow\infty} \bigl\Vert K\bigl(z^{k_{i}}\bigr) \bigr\Vert =0, $$

that is, $K(\hat{z})=0$. By noting that the equality in (17) can be rewritten as

$$\frac{z^{k_{i}}-w^{k_{i}}}{\beta_{k_{i}}}-K^{*}K\bigl(z^{k_{i}}\bigr)\in N_{S} \bigl(w^{k_{i}}\bigr), $$

and that the graph of the maximal monotone operator $N_{S}$ is weakly-strongly closed, and by passing to the limit in the last inclusions, we obtain, from (44) and (45), that

$$\hat{z} \in S. $$

Hence $\hat{z} \in\Gamma$. Now we can apply Lemma 2.3 to $D:=\Gamma$ to get that the full sequence $(z^{k})$ converges weakly to a point in Γ. This completes the proof. □

#### Remark 3.4

By using Remark 3.1, the contraction inequality (41) can be rewritten as follows:

46 $$ \bigl\Vert x^{k+1}_{I}-x^{*} \bigr\Vert ^{2}+ \bigl\Vert y^{k+1}_{I}-y^{*} \bigr\Vert ^{2} \leq \bigl\Vert x^{k}-x^{*} \bigr\Vert ^{2}+ \bigl\Vert y^{k}-y^{*} \bigr\Vert ^{2}-\gamma(2-\gamma) \rho_{k}^{2}\bigl( \Vert c_{k} \Vert ^{2}+ \Vert d_{k} \Vert ^{2}\bigr). $$

It is obvious that the $\rho_{k}$ in (31) is larger than that in (10). Let $\gamma=1$. Comparing (46) and (11), we claim that Algorithm 3.1(I) has a better contraction property than Algorithm 1.2.

#### Theorem 3.2

*Let* $(z^{k})$ *be generated by Algorithm* 3.1(II). *Assume that* Γ *is nonempty*. *Then we have*

47 $$ \bigl\Vert z^{k+1}_{\mathrm{II}}-z^{*} \bigr\Vert ^{2}\leq \bigl\Vert z^{k}-z^{*} \bigr\Vert ^{2}- \gamma(2-\gamma)\rho_{k}^{2} \bigl\Vert d \bigl(z^{k},w^{k}\bigr) \bigr\Vert ^{2}- \bigl\Vert z^{k+1}_{\mathrm{I}}-z^{k+1}_{\mathrm{II}} \bigr\Vert ^{2},\quad \forall z^{*}\in \Gamma. $$

*Furthermore*, $(z^{k})$ *converges weakly to a solution of SEP* (1).

#### Proof

Let $z^{*}\in\Gamma$, that is, $z^{*}\in S$, and $K(z^{*})=0$. Using Lemma 2.2(ii), we have

48 $$\begin{aligned} \bigl\Vert z^{k+1}_{\mathrm{II}}-z^{*} \bigr\Vert ^{2}& \leq \bigl\Vert z^{k}-\gamma\beta_{k}\rho_{k} K^{*}K \bigl(w^{k}\bigr)-z^{*} \bigr\Vert ^{2}- \bigl\Vert z^{k}-\gamma\beta_{k}\rho_{k} K^{*}K \bigl(w^{k}\bigr)-z^{k+1}_{\mathrm{II}} \bigr\Vert ^{2} \\ &= \bigl\Vert z^{k}-z^{*} \bigr\Vert ^{2}- \bigl\Vert z^{k}-z^{k+1}_{\mathrm{II}} \bigr\Vert ^{2}-2 \gamma\beta_{k}\rho_{k}\bigl\langle z^{k+1}_{\mathrm{II}}-z^{*},K^{*}K \bigl(w^{k}\bigr)\bigr\rangle . \end{aligned}$$

By setting $z=z^{k+1}_{\mathrm{II}}$ in (33), we get

49 $$\begin{aligned} &{-}2\gamma\beta_{k}\rho_{k}\bigl\langle z^{k+1}_{\mathrm{II}}-w^{k},K^{*}K\bigl(w^{k}\bigr) \bigr\rangle \\ &\quad \leq-2\gamma\rho_{k}\bigl\langle z^{k+1}_{\mathrm{II}}-w^{k},d \bigl(z^{k},w^{k}\bigr)\bigr\rangle \\ &\quad =-2\gamma\rho_{k}\bigl\langle z^{k}-w^{k},d \bigl(z^{k},w^{k}\bigr)\bigr\rangle -2\gamma\rho_{k} \bigl\langle z^{k+1}_{\mathrm{II}}-z^{k},d \bigl(z^{k},w^{k}\bigr)\bigr\rangle . \end{aligned}$$

It holds

50 $$\begin{aligned} -2\gamma\rho_{k}\bigl\langle z^{k+1}_{\mathrm{II}}-z^{k},d \bigl(z^{k},w^{k}\bigr)\bigr\rangle =&- \bigl\Vert z^{k}-z^{k+1}_{\mathrm{II}}-\gamma\rho_{k} d \bigl(z^{k},w^{k}\bigr) \bigr\Vert ^{2} \\ &{} + \bigl\Vert z^{k}-z^{k+1}_{\mathrm{II}} \bigr\Vert ^{2}+\gamma^{2}\rho_{k}^{2} \bigl\Vert d\bigl(z^{k},w^{k}\bigr) \bigr\Vert ^{2}. \end{aligned}$$

Substituting (50) in the right-hand side of (49) and using $z^{k}-\gamma\rho_{k} d(z^{k},w^{k})=z^{k+1}_{\mathrm{I}}$, we obtain

51 $$\begin{aligned} &{-}2\gamma\beta_{k}\rho_{k}\bigl\langle z^{k+1}_{\mathrm{II}}-w^{k},K^{*}K\bigl(w^{k}\bigr) \bigr\rangle \\ &\quad \leq-2\gamma\rho_{k}\bigl\langle z^{k}-w^{k}, d\bigl(z^{k},w^{k}\bigr)\bigr\rangle - \bigl\Vert z^{k+1}_{\mathrm{I}}-z^{k+1}_{\mathrm{II}} \bigr\Vert ^{2} \\ &\qquad {}+ \bigl\Vert z^{k}-z^{k+1}_{\mathrm{II}} \bigr\Vert ^{2}+\gamma^{2}\rho_{k}^{2} \bigl\Vert d\bigl(z^{k},w^{k}\bigr) \bigr\Vert ^{2}. \end{aligned}$$

From (40), we get

52 $$ -2\gamma\beta_{k}\rho_{k}\bigl\langle w^{k}-w^{*},K^{*}K\bigl(w^{k}\bigr)\bigr\rangle =-2\gamma \beta_{k}\rho_{k} \bigl\Vert K\bigl(w^{k}\bigr) \bigr\Vert ^{2}. $$

So, adding (51) and (52) and using the definition of $\rho_{k}$, we obtain

53 $$\begin{aligned} &{-}2\gamma\beta_{k}\rho_{k}\bigl\langle z^{k+1}_{\mathrm{II}}-z^{*},K^{*}K\bigl(w^{k}\bigr)\bigr\rangle \\ &\quad \leq-2\gamma\rho_{k}\bigl(\bigl\langle z^{k}-w^{k}, d\bigl(z^{k},w^{k}\bigr)\bigr\rangle +\beta_{k} \bigl\Vert K\bigl(w^{k}\bigr) \bigr\Vert ^{2}\bigr) \\ &\qquad {} - \bigl\Vert z^{k+1}_{\mathrm{I}}-z^{k+1}_{\mathrm{II}} \bigr\Vert ^{2}+ \bigl\Vert z^{k}-z^{k+1}_{\mathrm{II}} \bigr\Vert ^{2}+\gamma ^{2}\rho_{k}^{2} \bigl\Vert d\bigl(z^{k},w^{k}\bigr) \bigr\Vert ^{2} \\ &\quad \leq-2\gamma\rho_{k}^{2} \bigl\Vert d \bigl(z^{k},w^{k}\bigr) \bigr\Vert ^{2}+ \gamma^{2}\rho_{k}^{2} \bigl\Vert d \bigl(z^{k},w^{k}\bigr) \bigr\Vert ^{2}- \bigl\Vert z^{k+1}_{\mathrm{I}}-z^{k+1}_{\mathrm{II}} \bigr\Vert ^{2}+ \bigl\Vert z^{k}-z^{k+1}_{\mathrm{II}} \bigr\Vert ^{2} \\ &\quad \leq-\gamma(2-\gamma)\rho_{k}^{2} \bigl\Vert d \bigl(z^{k},w^{k}\bigr) \bigr\Vert ^{2}- \bigl\Vert z^{k+1}_{\mathrm{I}}-z^{k+1}_{\mathrm{II}} \bigr\Vert ^{2}+ \bigl\Vert z^{k}-z^{k+1}_{\mathrm{II}} \bigr\Vert ^{2}. \end{aligned}$$

Adding (48) and (53), we obtain (47). Employing arguments which are similar to those used in the proof of Theorem 3.1, we obtain that the whole sequence $(z^{k})$ weakly converges to a solution of SEP (1), which completes proof. □

#### Remark 3.5

Comparing inequalities (41) and (47), we conclude that Algorithm 3.1(II) seems to have a better contraction property than Algorithm 3.1(I) since $z^{k+1}_{\mathrm{II}}$ is closer to $z^{*}$ than $z^{k+1}_{\mathrm{I}}$ when $z^{k}$ is the same.

### Semi-alternating projection algorithms

Inspired by Algorithm 2.2 in [15] and based on Algorithm 3.1, we present two semi-alternating projection algorithms, whose name comes from an alternating technique taken in the first step.

#### Algorithm 3.2

Given constants $\sigma_{0} >0$, $\alpha\in(0,1)$, $\theta\in(0,1)$ and $\rho\in(0,1)$, let $x^{0} \in H_{1}$ and $y^{0}\in H_{2}$ be taken arbitrarily.

For $k = 0,1,2,\ldots $ , compute

54 $$ \textstyle\begin{cases} u^{k}=P_{C}(x^{k}-\beta_{k} F(x^{k},y^{k})), \\ v^{k}=P_{Q}(y^{k}-\beta_{k} G(u^{k},y^{k})), \end{cases} $$

where $\beta_{k}$ is chosen to be the largest $\beta\in\{\sigma_{k},\sigma _{k}\alpha,\sigma_{k}\alpha^{2},\ldots \}$ satisfying

55 $$\begin{aligned}& \beta^{2}\bigl( \bigl\Vert F\bigl(x^{k},y^{k} \bigr)-F\bigl(u^{k},v^{k}\bigr) \bigr\Vert ^{2}+ \bigl\Vert G\bigl(u^{k},y^{k}\bigr)-G\bigl(u^{k},v^{k} \bigr) \bigr\Vert ^{2}\bigr) \\& \quad \leq \theta^{2}\bigl( \bigl\Vert x^{k}-u^{k} \bigr\Vert ^{2}+ \bigl\Vert y^{k}-v^{k} \bigr\Vert ^{2}\bigr). \end{aligned}$$

Compute next iterates $x^{k+1}$ and $y^{k+1}$ by

56 $$ \textstyle\begin{cases} x^{k+1}_{\mathrm{I}}=x^{k}-\gamma\rho_{k} c_{k}, \\ y^{k+1}_{\mathrm{I}}=y^{k}-\gamma\rho_{k} d_{k}, \end{cases} $$

or

57 $$ \textstyle\begin{cases} x^{k+1}_{\mathrm{II}}=P_{C}(x^{k}-\gamma\beta_{k}\rho_{k} F(u^{k},v^{k})), \\ y^{k+1}_{\mathrm{II}}=P_{Q}(y^{k}-\gamma\beta_{k}\rho_{k} G(u^{k},v^{k})), \end{cases} $$

where $\gamma\in[0,2)$,

58 $$ \textstyle\begin{cases} c_{k}:=(x^{k}-u^{k})-\beta_{k}(F(x^{k},y^{k})-F(u^{k},v^{k})); \\ d_{k}:=(y^{k}-v^{k})-\beta_{k}(G(u^{k},y^{k})-G(u^{k},v^{k})), \end{cases} $$

and

59 $$ \rho_{k}:=\frac{\langle x^{k}-u^{k}, c_{k}\rangle+\langle y^{k}-v^{k}, d_{k}\rangle +\beta_{k}\| Au^{k}-Bv^{k}\|^{2}}{\|c_{k}\|^{2}+\|d_{k}\|^{2}}. $$

If

60 $$\begin{aligned}& {\beta_{k}^{2}}\bigl( \bigl\Vert F\bigl(x^{k},y^{k} \bigr)-F\bigl(x^{k+1},y^{k+1}\bigr) \bigr\Vert ^{2}+ \bigl\Vert G\bigl(x^{k},y^{k}\bigr)-G\bigl(x^{k+1},y^{k+1} \bigr) \bigr\Vert ^{2}\bigr) \\& \quad \leq\rho^{2}\bigl({ \bigl\Vert x^{k}-x^{k+1} \bigr\Vert ^{2}+ \bigl\Vert y^{k}-y^{k+1} \bigr\Vert ^{2}}\bigr), \end{aligned}$$

then set $\sigma_{k}=\sigma_{0}$; otherwise, set $\sigma_{k}=\beta_{k}$.

For convenience, we call the projection algorithms which use update forms (56) and (57) Algorithm 3.2(I) and Algorithm 3.2(II), respectively.

#### Remark 3.6

By the definitions of $c_{k}$ and $d_{k}$ in (58), the projection equation (54) can be written as

$$\textstyle\begin{cases} u^{k}=P_{C}(u^{k}-(\beta_{k} F(u^{k},v^{k})-c_{k})), \\ v^{k}=P_{Q}(v^{k}-(\beta_{k} G(u^{k},v^{k})-d_{k})). \end{cases} $$

So, from Lemma 2.1 we have

61 $$ \textstyle\begin{cases} \langle x-u^{k},\beta_{k} F(u^{k},v^{k})-c_{k}\rangle\geq0, \quad \forall x \in C, \\ \langle y-v^{k},\beta_{k} G(u^{k},v^{k})-d_{k}\rangle\geq0, \quad \forall y \in Q. \end{cases} $$

#### Lemma 3.4

*The search rule* (55) *is well defined*. *Besides* $\underline{\beta }^{*}\leq\beta_{k}\leq\sigma_{0}$, *where*

62 $$ \underline{\beta}^{*}=\min\biggl\{ \sigma_{0}, \frac{\alpha\theta}{\sqrt{2}\|A\|^{2}}, \frac{\alpha\theta}{\|B\|\sqrt{2(\|A\|^{2}+\|B\|^{2})}}\biggr\} . $$

#### Proof

Obviously, $\beta_{k}\leq\sigma_{k}\leq\sigma_{0}$. In the latter case, we know that $\beta_{k}/\alpha$ must violate inequality (55), that is,

$$ \begin{aligned} &\beta^{2}/\alpha^{2}\bigl( \bigl\Vert F \bigl(x^{k},y^{k}\bigr)-F\bigl(u^{k},v^{k} \bigr) \bigr\Vert ^{2}+ \bigl\Vert G\bigl(u^{k},y^{k} \bigr)-G\bigl(u^{k},v^{k}\bigr) \bigr\Vert ^{2} \bigr) \\ &\quad \geq\theta^{2}\bigl( \bigl\Vert x^{k}-u^{k} \bigr\Vert ^{2}+ \bigl\Vert y^{k}-v^{k} \bigr\Vert ^{2}\bigr). \end{aligned} $$

On the other hand, we have

$$\begin{aligned} & \bigl\Vert F\bigl(x^{k},y^{k}\bigr)-F \bigl(u^{k},v^{k}\bigr) \bigr\Vert ^{2}+ \bigl\Vert G\bigl(u^{k},y^{k}\bigr)-G\bigl(u^{k},v^{k} \bigr) \bigr\Vert ^{2} \\ &\quad = \bigl\Vert A^{*}\bigl(Ax^{k}-By^{k}\bigr)-A^{*} \bigl(Au^{k}-Bv^{k}\bigr) \bigr\Vert ^{2}+ \bigl\Vert B^{*}\bigl(By^{k}-Au^{k}\bigr)-B^{*}\bigl(Bv^{k}-Au^{k} \bigr) \bigr\Vert ^{2} \\ &\quad \leq \Vert A \Vert ^{2}\bigl( \bigl\Vert Ax^{k}-Au^{k} \bigr\Vert + \bigl\Vert By^{k}-Bv^{k} \bigr\Vert \bigr)^{2}+ \Vert B \Vert ^{4} \bigl\Vert y^{k}-v^{k} \bigr\Vert ^{2} \\ &\quad \leq2 \Vert A \Vert ^{2}\bigl( \Vert A \Vert ^{2} \bigl\Vert x^{k}-u^{k} \bigr\Vert ^{2}+ \Vert B \Vert ^{2} \bigl\Vert y^{k}-v^{k} \bigr\Vert ^{2}\bigr)+ \Vert B \Vert ^{4} \bigl\Vert y^{k}-v^{k} \bigr\Vert ^{2} \\ &\quad \leq2 \Vert A \Vert ^{4} \bigl\Vert x^{k}-u^{k} \bigr\Vert ^{2}+ \Vert B \Vert ^{2}\bigl(2 \Vert A \Vert ^{2}+ \Vert B \Vert ^{2}\bigr) \bigl\Vert y^{k}-v^{k} \bigr\Vert ^{2} \\ &\quad \leq\max\bigl\{ 2 \Vert A \Vert ^{4}+ \Vert B \Vert ^{2}\bigl(2 \Vert A \Vert ^{2}+ \Vert B \Vert ^{2}\bigr)\bigr\} \bigl( \bigl\Vert x^{k}-u^{k} \bigr\Vert ^{2}+ \bigl\Vert y^{k}-v^{k} \bigr\Vert ^{2}\bigr). \end{aligned}$$

So, we get (62). □

#### Lemma 3.5

*Let* $(x^{k},y^{k})$ *and* $(u^{k},v^{k})$ *be generated by Algorithm* 3.2 , *and let* $c_{k}$, $d_{k}$ *and* $\rho_{k}$ *be given by* (58) *and* (59), *respectively*. *Then we have*

$$\rho_{k}\geq\frac{1-\theta}{1+\theta^{2}}. $$

#### Proof

By the Cauchy-Schwarz inequality, we have

63 $$\begin{aligned} & \bigl\langle x^{k}-u^{k}, c_{k}\bigr\rangle + \bigl\langle y^{k}-v^{k}, d_{k}\bigr\rangle \\ &\quad = \bigl\Vert x^{k}-u^{k} \bigr\Vert ^{2}+ \bigl\Vert y^{k}-v^{k} \bigr\Vert ^{2}-\beta_{k}\bigl\langle x^{k}-u^{k},F \bigl(x^{k},y^{k}\bigr)-F\bigl(u^{k},v^{k} \bigr)\bigr\rangle \\ &\qquad {} -\beta_{k}\bigl\langle y^{k}-v^{k},G \bigl(u^{k},y^{k}\bigr)-G\bigl(u^{k},v^{k} \bigr)\bigr\rangle \\ &\quad \geq \bigl\Vert x^{k}-u^{k} \bigr\Vert ^{2}+ \bigl\Vert y^{k}-v^{k} \bigr\Vert ^{2} \\ &\qquad {} -\beta_{k}\bigl( \bigl\Vert x^{k}-u^{k} \bigr\Vert \bigl\Vert F\bigl(x^{k},y^{k}\bigr)-F \bigl(u^{k},v^{k}\bigr) \bigr\Vert + \bigl\Vert y^{k}-v^{k} \bigr\Vert \bigl\Vert G\bigl(u^{k},y^{k} \bigr)-G\bigl(u^{k},v^{k}\bigr) \bigr\Vert \bigr). \end{aligned}$$

It holds

64 $$\begin{aligned} & \beta_{k}^{2}\bigl( \bigl\Vert x^{k}-u^{k} \bigr\Vert \bigl\Vert F\bigl(x^{k},y^{k}\bigr)-F \bigl(u^{k},v^{k}\bigr) \bigr\Vert + \bigl\Vert y^{k}-v^{k} \bigr\Vert \bigl\Vert G\bigl(u^{k},y^{k} \bigr)-G\bigl(u^{k},v^{k}\bigr) \bigr\Vert \bigr)^{2} \\ &\quad =\beta_{k}^{2}\bigl( \bigl\Vert x^{k}-u^{k} \bigr\Vert ^{2} \bigl\Vert F \bigl(x^{k},y^{k}\bigr)-F\bigl(u^{k},v^{k} \bigr) \bigr\Vert ^{2}+ \bigl\Vert y^{k}-v^{k} \bigr\Vert ^{2} \bigl\Vert G\bigl(u^{k},y^{k} \bigr)-G\bigl(u^{k},v^{k}\bigr) \bigr\Vert ^{2} \\ &\qquad {} +2 \bigl\Vert x^{k}-u^{k} \bigr\Vert \bigl\Vert F\bigl(x^{k},y^{k}\bigr)-F\bigl(u^{k},v^{k} \bigr) \bigr\Vert \bigl\Vert y^{k}-v^{k} \bigr\Vert \bigl\Vert G\bigl(u^{k},y^{k}\bigr)-G\bigl(u^{k},v^{k} \bigr) \bigr\Vert \bigr) \\ &\quad \leq\beta_{k}^{2}\bigl( \bigl\Vert x^{k}-u^{k} \bigr\Vert ^{2} \bigl\Vert F \bigl(x^{k},y^{k}\bigr)-F\bigl(u^{k},v^{k} \bigr) \bigr\Vert ^{2}+ \bigl\Vert y^{k}-v^{k} \bigr\Vert ^{2} \bigl\Vert G\bigl(u^{k},y^{k} \bigr)-G\bigl(u^{k},v^{k}\bigr) \bigr\Vert ^{2} \\ &\qquad {} + \bigl\Vert x^{k}-u^{k} \bigr\Vert ^{2} \bigl\Vert G\bigl(u^{k},y^{k}\bigr)-G \bigl(u^{k},v^{k}\bigr) \bigr\Vert ^{2}+ \bigl\Vert y^{k}-v^{k} \bigr\Vert ^{2} \bigl\Vert F \bigl(x^{k},y^{k}\bigr)-F\bigl(u^{k},v^{k} \bigr) \bigr\Vert ^{2}\bigr) \\ &\quad =\beta_{k}^{2}\bigl( \bigl\Vert F \bigl(x^{k},y^{k}\bigr)-F\bigl(u^{k},v^{k} \bigr) \bigr\Vert ^{2}+ \bigl\Vert G\bigl(u^{k},y^{k} \bigr)-G\bigl(u^{k},v^{k}\bigr) \bigr\Vert ^{2} \bigr) \bigl( \bigl\Vert x^{k}-u^{k} \bigr\Vert ^{2}+ \bigl\Vert y^{k}-v^{k} \bigr\Vert ^{2}\bigr) \\ &\quad \leq\theta^{2}\bigl( \bigl\Vert x^{k}-u^{k} \bigr\Vert ^{2}+ \bigl\Vert y^{k}-v^{k} \bigr\Vert ^{2}\bigr)^{2}. \end{aligned}$$

So, we obtain

65 $$\begin{aligned} \begin{aligned}[b] & \bigl\langle x^{k}-u^{k}, c_{k}\bigr\rangle + \bigl\langle y^{k}-v^{k}, d_{k}\bigr\rangle \\ &\quad \geq \bigl\Vert x^{k}-u^{k} \bigr\Vert ^{2}+ \bigl\Vert y^{k}-v^{k} \bigr\Vert ^{2}-\theta \bigl( \bigl\Vert x^{k}-u^{k} \bigr\Vert ^{2}+ \bigl\Vert y^{k}-v^{k} \bigr\Vert ^{2}\bigr) \\ &\quad =(1-\theta) \bigl( \bigl\Vert x^{k}-u^{k} \bigr\Vert ^{2}+ \bigl\Vert y^{k}-v^{k} \bigr\Vert ^{2}\bigr). \end{aligned} \end{aligned}$$

From the definition of *F* and *G*, we have

66 $$\begin{aligned} & \Vert c_{k} \Vert ^{2}+ \Vert d_{k} \Vert ^{2} \\ &\quad = \bigl\Vert x^{k}-u^{k} \bigr\Vert ^{2}+ \bigl\Vert y^{k}-v^{k} \bigr\Vert ^{2} \\ &\qquad {} +\beta_{k}^{2}\bigl( \bigl\Vert F \bigl(x^{k},y^{k}\bigr)-F\bigl(u^{k},v^{k} \bigr) \bigr\Vert ^{2}+ \bigl\Vert G\bigl(u^{k},y^{k} \bigr)-G\bigl(u^{k},v^{k}\bigr) \bigr\Vert ^{2} \bigr) \\ &\qquad {} -2\beta_{k}\bigl(\bigl\langle x^{k}-u^{k},F \bigl(x^{k},y^{k}\bigr)-F\bigl(u^{k},v^{k} \bigr)\bigr\rangle +\bigl\langle y^{k}-v^{k},G \bigl(u^{k},y^{k}\bigr)-G\bigl(u^{k},v^{k} \bigr)\bigr\rangle \bigr) \\ &\quad \leq \bigl\Vert x^{k}-u^{k} \bigr\Vert ^{2}+ \bigl\Vert y^{k}-v^{k} \bigr\Vert ^{2} +\theta^{2}\bigl( \bigl\Vert x^{k}-u^{k} \bigr\Vert ^{2}+ \bigl\Vert y^{k}-v^{k} \bigr\Vert ^{2}\bigr) \\ &\qquad {} -2\beta_{k}\bigl(\bigl\langle A\bigl(x^{k}-u^{k} \bigr),A\bigl(x^{k}-u^{k}\bigr)-B\bigl(y^{k}-v^{k} \bigr)\bigr\rangle \\ &\qquad {}-\bigl\langle B\bigl(y^{k}-v^{k}\bigr),A \bigl(u^{k}-u^{k}\bigr)-B\bigl(y^{k}-v^{k} \bigr)\bigr\rangle \bigr) \\ &\quad \leq \bigl\Vert x^{k}-u^{k} \bigr\Vert ^{2}+ \bigl\Vert y^{k}-v^{k} \bigr\Vert ^{2} +\theta^{2}\bigl( \bigl\Vert x^{k}-u^{k} \bigr\Vert ^{2}+ \bigl\Vert y^{k}-v^{k} \bigr\Vert ^{2}\bigr) \\ &\qquad {} -2\beta_{k}\bigl( \bigl\Vert A\bigl(x^{k}-u^{k} \bigr) \bigr\Vert ^{2}-\bigl\langle A\bigl(x^{k}-u^{k} \bigr),B\bigl(y^{k}-v^{k}\bigr)\bigr\rangle + \bigl\Vert B \bigl(y^{k}-v^{k}\bigr) \bigr\Vert ^{2} \bigr). \end{aligned}$$

Since

$$\begin{aligned} & {-}2\beta_{k}\bigl( \bigl\Vert A\bigl(x^{k}-u^{k} \bigr) \bigr\Vert ^{2}-\bigl\langle A\bigl(x^{k}-u^{k} \bigr),B\bigl(y^{k}-v^{k}\bigr)\bigr\rangle + \bigl\Vert B \bigl(y^{k}-v^{k}\bigr) \bigr\Vert ^{2}\bigr) \\ &\quad \leq-2\beta_{k}\bigl( \bigl\Vert A\bigl(x^{k}-u^{k} \bigr) \bigr\Vert ^{2}- \bigl\Vert A\bigl(x^{k}-u^{k} \bigr) \bigr\Vert \bigl\Vert B\bigl(y^{k}-v^{k}\bigr) \bigr\Vert + \bigl\Vert B\bigl(y^{k}-v^{k}\bigr) \bigr\Vert ^{2}\bigr) \\ &\quad \leq-2\beta_{k}\biggl( \bigl\Vert A\bigl(x^{k}-u^{k} \bigr) \bigr\Vert ^{2}-\frac{1}{2}\bigl( \bigl\Vert A \bigl(x^{k}-u^{k}\bigr) \bigr\Vert ^{2}+ \bigl\Vert B\bigl(y^{k}-v^{k}\bigr) \bigr\Vert ^{2} \bigr) + \bigl\Vert B\bigl(y^{k}-v^{k}\bigr) \bigr\Vert ^{2}\biggr) \\ &\quad \leq-\beta_{k}\bigl( \bigl\Vert A\bigl(x^{k}-u^{k} \bigr) \bigr\Vert ^{2}+ \bigl\Vert B\bigl(y^{k}-v^{k} \bigr) \bigr\Vert ^{2}\bigr), \end{aligned}$$

by (66), we get

67 $$\begin{aligned} & \Vert c_{k} \Vert ^{2}+ \Vert d_{k} \Vert ^{2} \\ &\quad \leq\bigl(1+\theta^{2}\bigr) \bigl( \bigl\Vert x^{k}-u^{k} \bigr\Vert ^{2}+ \bigl\Vert y^{k}-v^{k} \bigr\Vert ^{2}\bigr)- \beta_{k}\bigl( \bigl\Vert A\bigl(x^{k}-u^{k} \bigr) \bigr\Vert ^{2}+ \bigl\Vert B\bigl(y^{k}-v^{k} \bigr) \bigr\Vert ^{2}\bigr) \\ &\quad \leq\bigl(1+\theta^{2}\bigr) \bigl( \bigl\Vert x^{k}-u^{k} \bigr\Vert ^{2}+ \bigl\Vert y^{k}-v^{k} \bigr\Vert ^{2}\bigr). \end{aligned}$$

Combining (65) and (67), we complete the proof. □

#### Lemma 3.6

*Let* $(x^{k},y^{k})$ *and* $(u^{k},v^{k})$ *be generated by Algorithm* 3.2, *and let* $c_{k}$ *and* $d_{k}$ *be given by* (58). *Then*, *for all* $(x^{*},y^{*})\in\Gamma$, *we have*

$$\bigl\langle x^{k}-x^{*}, c_{k}\bigr\rangle +\bigl\langle y^{k}-y^{*}, d_{k}\bigr\rangle \geq\rho_{k}\bigl( \Vert c_{k} \Vert ^{2}+ \Vert d_{k} \Vert ^{2}\bigr). $$

#### Proof

Take arbitrarily $(x^{*},y^{*})\in\Gamma$, that is, $x^{*}\in C, y^{*}\in Q$, and $Ax^{*}=By^{*}$. By setting $(x,y)=(x^{*},y^{*})$ in (61), we get

$$\textstyle\begin{cases} \langle x^{*}-u^{k},\beta_{k} F(u^{k},v^{k})-c_{k}\rangle\geq0, \\ \langle y^{*}-v^{k},\beta_{k} G(u^{k},v^{k})-d_{k}\rangle\geq0, \end{cases} $$

which implies that

$$\textstyle\begin{cases} \langle u^{k}-x^{*},c_{k}\rangle\geq\beta_{k} \langle u^{k}-x^{*},F(u^{k},v^{k})\rangle, \\ \langle v^{k}-y^{*},d_{k}\rangle\geq\beta_{k} \langle v^{k}-y^{*},G(u^{k},v^{k})\rangle. \end{cases} $$

By the definition of *F* and *G*, we have

68 $$\begin{aligned} & \bigl\langle u^{k}-x^{*},F\bigl(u^{k},v^{k}\bigr) \bigr\rangle +\bigl\langle v^{k}-y^{*},G\bigl(u^{k},v^{k} \bigr)\bigr\rangle \\ &\quad =\bigl\langle u^{k}-x^{*},A^{*}\bigl(Au^{k}-Bv^{k} \bigr)\bigr\rangle +\bigl\langle v^{k}-y^{*},B^{*}\bigl(Bv^{k}-Au^{k} \bigr)\bigr\rangle \\ &\quad =\bigl\langle Au^{k}-Ax^{*},Au^{k}-Bv^{k} \bigr\rangle +\bigl\langle Bv^{k}-By^{*},Bv^{k}-Au^{k} \bigr\rangle \\ &\quad =\bigl\langle Au^{k}-Bv^{k}-\bigl(Ax^{*}-By^{*} \bigr),Au^{k}-Bv^{k}\bigr\rangle \\ &\quad = \bigl\Vert Au^{k}-Bv^{k} \bigr\Vert ^{2}, \end{aligned}$$

and

$$\begin{aligned} & \bigl\langle x^{k}-x^{*},c_{k}\bigr\rangle +\bigl\langle y^{k}-y^{*},d_{k}\bigr\rangle \\ &\quad =\bigl\langle x^{k}-u^{k},c_{k}\bigr\rangle +\bigl\langle y^{k}-v^{k},d_{k}\bigr\rangle + \bigl\langle u^{k}-x^{*},c_{k}\bigr\rangle +\bigl\langle v^{k}-y^{*},d_{k}\bigr\rangle \\ &\quad \geq\bigl\langle x^{k}-u^{k},c_{k}\bigr\rangle +\bigl\langle y^{k}-v^{k},d_{k}\bigr\rangle + \beta_{k} \bigl\Vert Au^{k}-Bv^{k} \bigr\Vert ^{2} \\ &\quad =\rho_{k}\bigl( \Vert c_{k} \Vert ^{2}+ \Vert d_{k} \Vert ^{2}\bigr). \end{aligned}$$

So, we complete the proof. □

#### Theorem 3.3

*Let* $(x^{k},y^{k})$ *be generated by Algorithm* 3.2(I). *If* Γ *is nonempty*, *then we have*

69 $$\begin{aligned}& \bigl\Vert x^{k+1}_{\mathrm{I}}-x^{*} \bigr\Vert ^{2}+ \bigl\Vert y^{k+1}_{\mathrm{I}}-y^{*} \bigr\Vert ^{2} \\& \quad \leq \bigl\Vert x^{k}-x^{*} \bigr\Vert ^{2}+ \bigl\Vert y^{k}-y^{*} \bigr\Vert ^{2}-(2-\gamma)\gamma \rho_{k}^{2}\bigl( \Vert c_{k} \Vert ^{2}+ \Vert d_{k} \Vert ^{2}\bigr), \quad \forall\bigl(x^{*},y^{*}\bigr)\in\Gamma, \end{aligned}$$

*and* $(x^{k},y^{k})$ *converges weakly to a solution of SEP* (1).

#### Proof

Let $(x^{*},y^{*})\in\Gamma$, that is, $x^{*}\in C$, $y^{*}\in Q$, and $Ax^{*}=By^{*}$. Then we have

$$\begin{aligned} \bigl\Vert x^{k+1}_{\mathrm{I}}-x^{*} \bigr\Vert ^{2}&= \bigl\Vert x^{k}-\gamma\rho_{k} c_{k}-x^{*} \bigr\Vert ^{2} \\ &= \bigl\Vert x^{k}-x^{*} \bigr\Vert ^{2}+ \gamma^{2}\rho_{k}^{2} \Vert c_{k} \Vert ^{2}-2\gamma\rho_{k}\bigl\langle x^{k}-x^{*},c_{k} \bigr\rangle . \end{aligned}$$

Similarly, we get

$$\bigl\Vert y^{k+1}_{\mathrm{I}}-y^{*} \bigr\Vert ^{2} = \bigl\Vert y^{k}-y^{*} \bigr\Vert ^{2}+\gamma^{2} \rho_{k}^{2} \Vert d_{k} \Vert ^{2}-2 \gamma\rho_{k}\bigl\langle y^{k}-y^{*},d_{k}\bigr\rangle . $$

Adding the above inequalities and using Lemma 3.6, we have

70 $$\begin{aligned} & \bigl\Vert x^{k+1}_{\mathrm{I}}-x^{*} \bigr\Vert ^{2}+ \bigl\Vert y^{k+1}_{\mathrm{I}}-y^{*} \bigr\Vert ^{2} \\ &\quad = \bigl\Vert x^{k}-x^{*} \bigr\Vert ^{2}+ \bigl\Vert y^{k}-y^{*} \bigr\Vert ^{2}+\gamma^{2} \rho_{k}^{2}\bigl( \Vert c_{k} \Vert ^{2}+ \Vert d_{k} \Vert ^{2}\bigr) \\ &\qquad {} -2\gamma\rho_{k}\bigl\langle x^{k}-x^{*},c_{k} \bigr\rangle -2\gamma\rho_{k}\bigl\langle y^{k}-y^{*},d_{k} \bigr\rangle \\ &\quad \leq \bigl\Vert x^{k}-x^{*} \bigr\Vert ^{2}+ \bigl\Vert y^{k}-y^{*} \bigr\Vert ^{2}-(2-\gamma)\gamma \rho_{k}^{2}\bigl( \Vert c_{k} \Vert ^{2}+ \Vert d_{k} \Vert ^{2} \bigr), \end{aligned}$$

which yields (69). Since $\gamma\in(0,2)$, (70) implies that the sequence $\| x^{k}-x^{*}\|^{2}+\|y^{k}-y^{*}\|^{2}$ is decreasing and thus converges. Moreover, $(x^{k})$ and $(y^{k})$ are bounded. This implies that

71 $$ \lim_{k\rightarrow\infty}\rho_{k}^{2} \bigl(\|c_{k}\|^{2}+\| d_{k}\|^{2} \bigr)=0. $$

From the definition of $\rho_{k}$, Lemmas 3.4 and 3.5, we have

$$\begin{aligned} & \rho_{k}^{2}\bigl( \Vert c_{k} \Vert ^{2}+ \Vert d_{k} \Vert ^{2}\bigr) \\ &\quad =\rho_{k}\bigl(\bigl\langle x^{k}-u^{k},c_{k} \bigr\rangle +\bigl\langle y^{k}-v^{k}, d_{k}\bigr\rangle +\beta _{k} \bigl\Vert Au^{k}-Bv^{k} \bigr\Vert ^{2}\bigr) \\ &\quad \geq\rho_{k} \bigl[(1-\theta) \bigl( \bigl\Vert x^{k}-u^{k} \bigr\Vert ^{2}+ \bigl\Vert y^{k}-v^{k} \bigr\Vert ^{2}\bigr)+ \beta_{k} \bigl\Vert Au^{k}-Bv^{k} \bigr\Vert ^{2} \bigr] \\ &\quad \geq\frac{(1-\theta)^{2}}{1+\theta^{2}}\bigl( \bigl\Vert x^{k}-u^{k} \bigr\Vert ^{2}+ \bigl\Vert y^{k}-v^{k} \bigr\Vert ^{2}\bigr)+\frac {1-\theta}{1+\theta^{2}}\underline{\beta} \bigl\Vert Au^{k}-Bv^{k} \bigr\Vert ^{2}, \end{aligned}$$

which implies

$$\bigl\Vert x^{k}-u^{k} \bigr\Vert ^{2}+ \bigl\Vert y^{k}-v^{k} \bigr\Vert ^{2}\leq \frac{1+\theta^{2}}{(1-\theta)^{2}}\rho _{k}^{2}\bigl( \Vert c_{k} \Vert ^{2}+ \Vert d_{k} \Vert ^{2}\bigr) $$

and

$$\bigl\Vert Au^{k}-Bv^{k} \bigr\Vert ^{2}\leq \frac{1+\theta^{2}}{(1-\theta)\underline{\beta}}\rho _{k}^{2}\bigl( \Vert c_{k} \Vert ^{2}+ \Vert d_{k} \Vert ^{2}\bigr). $$

Using (71), we get

72 $$ \lim_{k\rightarrow\infty} \bigl\Vert x^{k}-u^{k} \bigr\Vert + \bigl\Vert y^{k}-v^{k} \bigr\Vert =0, $$

and

73 $$ \lim_{k\rightarrow\infty} \bigl\Vert Au^{k}-Bv^{k} \bigr\Vert =0. $$

Hence, we get

$$\lim_{k\rightarrow\infty} \bigl\Vert Ax^{k}-By^{k} \bigr\Vert =0. $$

Let $(\hat{x},\hat{y})\in\omega_{w}(x^{k},y^{k})$, then there exist two subsequences $(x^{k_{i}})$ and $(y^{k_{i}})$ of $(x^{k})$ and $(y^{k})$ which converge weakly to *x̂* and *ŷ*, respectively. From (72), it follows that $(u^{k_{i}})$ and $(v^{k_{i}})$ also converge weakly to *x̂* and *ŷ*, respectively. We will show that $(\hat{x},\hat{y})$ is a solution of SEP (1). The weak convergence of $(Ax^{k_{i}}-By^{k_{i}})$ to $A\hat{x}-B\hat{y}$ and the lower semicontinuity of the squared norm imply that

$$\Vert A\hat{x}-B\hat{y} \Vert \leq\liminf_{i\rightarrow\infty} \bigl\Vert Ax^{k_{i}}-By^{k_{i}} \bigr\Vert =0, $$

that is, $A\hat{x}=B\hat{y}$. By noting that the two equalities in (54) can be rewritten as

$$\textstyle\begin{cases} \frac{x^{k_{i}}-u^{k_{i}}}{\beta_{k_{i}}}-A^{*}(Au^{k_{i}}-Bv^{k_{i}})\in N_{C}(u^{k_{i}}), \\ \frac{y^{k_{i}}-v^{k_{i}}}{\beta_{k_{i}}}-B^{*}(Bv^{k_{i}}-Au^{k_{i}})\in N_{Q}(v^{k_{i}}), \end{cases} $$

and that the graphs of the maximal monotone operators $N_{C}$ and $N_{Q}$ are weakly-strongly closed, and by passing to the limit in the last inclusions, we obtain, from (72) and (73), that

$$\hat{x} \in C, \qquad \hat{y} \in Q. $$

Hence $(\hat{x},\hat{y}) \in\Gamma$.

Now we can apply Lemma 2.3 to $D:=\Gamma$ to get that the full sequence $(x^{k},y^{k})$ converges weakly to a point in Γ. This completes the proof. □

#### Remark 3.7

Employing arguments which are similar to those used in Remark 3.4, comparing (69) and (2.48) in [15], we conclude that Algorithm 3.2(I) has a better contraction property than the hybrid alternating CQ-algorithm in [15].

#### Theorem 3.4

*Let* $(x^{k},y^{k})$ *be generated by Algorithm* 3.2(II). *If* Γ *is nonempty*, *then we have*

74 $$\begin{aligned} \bigl\Vert x^{k+1}_{\mathrm{II}}-x^{*} \bigr\Vert ^{2}+ \bigl\Vert y^{k+1}_{\mathrm{II}}-y^{*} \bigr\Vert ^{2} \leq& \bigl\Vert x^{k}-x^{*} \bigr\Vert ^{2}+ \bigl\Vert y^{k}-y^{*} \bigr\Vert ^{2}-\gamma(2-\gamma) \rho_{k}^{2}\bigl( \Vert c_{k} \Vert ^{2}+ \Vert d_{k} \Vert ^{2}\bigr) \\ &{} - \bigl\Vert x^{k+1}_{\mathrm{I}}-x^{k+1}_{\mathrm{II}} \bigr\Vert ^{2}- \bigl\Vert y^{k+1}_{\mathrm{I}}-y^{k+1}_{\mathrm{II}} \bigr\Vert ^{2}, \end{aligned}$$

*and* $(x^{k},y^{k})$ *converges weakly to a solution of SEP* (1).

#### Proof

Let $(x^{*},y^{*})\in\Gamma$, that is, $x^{*}\in C$, $y^{*}\in Q$, and $Ax^{*}=By^{*}$. Using Lemma 2.2(ii), we have

$$\begin{aligned} \bigl\Vert x^{k+1}_{\mathrm{II}}-x^{*} \bigr\Vert ^{2}& \leq \bigl\Vert x^{k}-\gamma\beta_{k}\rho_{k} F \bigl(u^{k},v^{k}\bigr)-x^{*} \bigr\Vert ^{2}- \bigl\Vert x^{k}-\gamma\beta_{k}\rho_{k} F \bigl(u^{k},v^{k}\bigr)-x^{k+1}_{\mathrm{II}} \bigr\Vert ^{2} \\ &= \bigl\Vert x^{k}-x^{*} \bigr\Vert ^{2}- \bigl\Vert x^{k}-x^{k+1}_{\mathrm{II}} \bigr\Vert ^{2}-2 \gamma\beta_{k}\rho_{k}\bigl\langle x^{k+1}_{\mathrm{II}}-x^{*},F \bigl(u^{k},v^{k}\bigr)\bigr\rangle . \end{aligned}$$

Similarly, we get

$$\bigl\Vert y^{k+1}_{\mathrm{II}}-y^{*} \bigr\Vert ^{2} \leq \bigl\Vert y^{k}-y^{*} \bigr\Vert ^{2}- \bigl\Vert y^{k}-y^{k+1}_{\mathrm{II}} \bigr\Vert ^{2}-2 \gamma\beta_{k}\rho_{k}\bigl\langle y^{k+1}_{\mathrm{II}}-y^{*},G \bigl(u^{k},v^{k}\bigr)\bigr\rangle . $$

Adding the above inequalities, we obtain

75 $$\begin{aligned} & \bigl\Vert x^{k+1}_{\mathrm{II}}-x^{*} \bigr\Vert ^{2}+ \bigl\Vert y^{k+1}_{\mathrm{II}}-y^{*} \bigr\Vert ^{2} \\ &\quad \leq \bigl\Vert x^{k}-x^{*} \bigr\Vert ^{2}+ \bigl\Vert y^{k}-y^{*} \bigr\Vert ^{2}- \bigl\Vert x^{k}-x^{k+1}_{\mathrm{II}} \bigr\Vert ^{2}- \bigl\Vert y^{k}-y^{k+1}_{\mathrm{II}} \bigr\Vert ^{2} \\ &\qquad {} -2\gamma\beta_{k}\rho_{k}\bigl\langle x^{k+1}_{\mathrm{II}}-x^{*},F\bigl(u^{k},v^{k}\bigr) \bigr\rangle -2\gamma\beta_{k}\rho_{k}\bigl\langle y^{k+1}_{\mathrm{II}}-y^{*},G\bigl(u^{k},v^{k}\bigr) \bigr\rangle . \end{aligned}$$

By setting $(x,y)=(x^{k+1}_{\mathrm{II}},y^{k+1}_{\mathrm{II}})$ in (61), we get

76 $$\begin{aligned} \begin{aligned}[b] & {-}2\gamma\beta_{k}\rho_{k}\bigl\langle x^{k+1}_{\mathrm{II}}-u^{k},F\bigl(u^{k},v^{k} \bigr)\bigr\rangle -2\gamma\beta_{k}\rho_{k}\bigl\langle y^{k+1}_{\mathrm{II}}-v^{k},G\bigl(u^{k},v^{k} \bigr)\bigr\rangle \\ &\quad \leq-2\gamma\rho_{k}\bigl\langle x^{k+1}_{\mathrm{II}}-u^{k},c_{k} \bigr\rangle -2\gamma\rho_{k}\bigl\langle y^{k+1}_{\mathrm{II}}-v^{k},d_{k} \bigr\rangle \\ &\quad =-2\gamma\rho_{k}\bigl(\bigl\langle x^{k}-u^{k},c_{k} \bigr\rangle +\bigl\langle y^{k}-v^{k},d_{k}\bigr\rangle \bigr)-2\gamma\rho_{k}\bigl(\bigl\langle x^{k+1}_{\mathrm{II}}-x^{k},c_{k} \bigr\rangle +\bigl\langle y^{k+1}_{\mathrm{II}}-y^{k},d_{k} \bigr\rangle \bigr). \end{aligned} \end{aligned}$$

It holds

77 $$ -2\gamma\rho_{k}\bigl\langle x^{k+1}_{\mathrm{II}}-x^{k},c_{k} \bigr\rangle =- \bigl\Vert x^{k}-x^{k+1}_{\mathrm{II}}- \gamma\rho_{k} c_{k} \bigr\Vert ^{2}+ \bigl\Vert x^{k}-x^{k+1}_{\mathrm{II}} \bigr\Vert ^{2}+ \gamma^{2}\rho_{k}^{2} \Vert c_{k} \Vert ^{2}. $$

Similarly, we get

78 $$ -2\gamma\rho_{k}\bigl\langle y^{k+1}_{\mathrm{II}}-y^{k},d_{k} \bigr\rangle =- \bigl\Vert y^{k}-y^{k+1}_{\mathrm{II}}-\gamma \rho_{k} d_{k} \bigr\Vert ^{2}+ \bigl\Vert y^{k}-y^{k+1}_{\mathrm{II}} \bigr\Vert ^{2}+ \gamma^{2}\rho_{k}^{2} \Vert d_{k} \Vert ^{2}. $$

Substituting (77) and (78) in the right-hand side of (76) and using $x^{k}-\gamma\rho_{k} c_{k}=x^{k+1}_{\mathrm{I}}$ and $y^{k}-\gamma\rho_{k} d_{k}=y^{k+1}_{\mathrm{I}}$, we obtain

79 $$\begin{aligned} & {-}2\gamma\beta_{k}\rho_{k}\bigl\langle x^{k+1}_{\mathrm{II}}-u^{k},F\bigl(u^{k},v^{k} \bigr)\bigr\rangle -2\gamma\beta_{k}\rho_{k}\bigl\langle y^{k+1}_{\mathrm{II}}-v^{k},G\bigl(u^{k},v^{k} \bigr)\bigr\rangle \\ &\quad \leq-2\gamma\rho_{k}\bigl(\bigl\langle x^{k}-u^{k},c_{k} \bigr\rangle +\bigl\langle y^{k}-v^{k},d_{k}\bigr\rangle \bigr) \\ &\qquad {} - \bigl\Vert x^{k+1}_{\mathrm{I}}-x^{k+1}_{\mathrm{II}} \bigr\Vert ^{2}+ \bigl\Vert x^{k}-x^{k+1}_{\mathrm{II}} \bigr\Vert ^{2}+\gamma^{2}\rho _{k}^{2} \Vert c_{k} \Vert ^{2} \\ &\qquad {} - \bigl\Vert y^{k+1}_{\mathrm{I}}-y^{k+1}_{\mathrm{II}} \bigr\Vert ^{2}+ \bigl\Vert y^{k}-y^{k+1}_{\mathrm{II}} \bigr\Vert ^{2}+\gamma^{2}\rho _{k}^{2} \Vert d_{k} \Vert ^{2}. \end{aligned}$$

From (68), we have

80 $$ \begin{aligned}[b] &{-}2\gamma\beta_{k}\rho_{k}\bigl\langle u^{k}-x^{*},F\bigl(u^{k},v^{k}\bigr)\bigr\rangle -2 \gamma\beta _{k}\rho_{k}\bigl\langle v^{k}-y^{*},G \bigl(u^{k},v^{k}\bigr)\bigr\rangle \\ &\quad =-2\gamma \beta_{k}\rho_{k} \bigl\Vert Au^{k}-Bv^{k} \bigr\Vert ^{2}. \end{aligned} $$

So, adding (79) and (80) and using the definition of $\rho_{k}$, we obtain

81 $$\begin{aligned} &{-}2\gamma\beta_{k}\rho_{k}\bigl\langle x^{k+1}_{\mathrm{II}}-x^{*},F\bigl(u^{k},v^{k}\bigr) \bigr\rangle -2\gamma\beta_{k}\rho_{k}\bigl\langle y^{k+1}_{\mathrm{II}}-y^{*},G\bigl(u^{k},v^{k}\bigr) \bigr\rangle \\ &\quad \leq-2\gamma\rho_{k}\bigl(\bigl\langle x^{k}-u^{k},c_{k} \bigr\rangle +\bigl\langle y^{k}-v^{k},d_{k}\bigr\rangle +\beta_{k} \bigl\Vert Au^{k}-Bv^{k} \bigr\Vert ^{2}\bigr) \\ &\qquad {} - \bigl\Vert x^{k+1}_{\mathrm{I}}-x^{k+1}_{\mathrm{II}} \bigr\Vert ^{2}+ \bigl\Vert x^{k}-x^{k+1}_{\mathrm{II}} \bigr\Vert ^{2}+\gamma^{2}\rho _{k}^{2} \Vert c_{k} \Vert ^{2} \\ &\qquad {} - \bigl\Vert y^{k+1}_{\mathrm{I}}-y^{k+1}_{\mathrm{II}} \bigr\Vert ^{2}+ \bigl\Vert y^{k}-y^{k+1}_{\mathrm{II}} \bigr\Vert ^{2}+\gamma^{2}\rho _{k}^{2} \Vert d_{k} \Vert ^{2} \\ &\quad \leq-2\gamma\rho_{k}^{2}\bigl( \Vert c_{k} \Vert ^{2}+ \Vert d_{k} \Vert ^{2}\bigr)+\gamma^{2}\rho_{k}^{2} \bigl( \Vert c_{k} \Vert ^{2}+ \Vert d_{k} \Vert ^{2}\bigr) \\ & \qquad {}- \bigl\Vert x^{k+1}_{\mathrm{I}}-x^{k+1}_{\mathrm{II}} \bigr\Vert ^{2}- \bigl\Vert y^{k+1}_{\mathrm{I}}-y^{k+1}_{\mathrm{II}} \bigr\Vert ^{2}+ \bigl\Vert x^{k}-x^{k+1}_{\mathrm{II}} \bigr\Vert ^{2} + \bigl\Vert y^{k}-y^{k+1}_{\mathrm{II}} \bigr\Vert ^{2} \\ &\quad \leq-\gamma(2-\gamma)\rho_{k}^{2}\bigl( \Vert c_{k} \Vert ^{2}+ \Vert d_{k} \Vert ^{2}\bigr) \\ &\qquad {} - \bigl\Vert x^{k+1}_{\mathrm{I}}-x^{k+1}_{\mathrm{II}} \bigr\Vert ^{2}- \bigl\Vert y^{k+1}_{\mathrm{I}}-y^{k+1}_{\mathrm{II}} \bigr\Vert ^{2}+ \bigl\Vert x^{k}-x^{k+1}_{\mathrm{II}} \bigr\Vert ^{2} + \bigl\Vert y^{k}-y^{k+1}_{\mathrm{II}} \bigr\Vert ^{2}. \end{aligned}$$

Adding (75) and (81), we obtain (74). Employing arguments which are similar to those used in the proof of Theorem 3.3, we obtain that the whole sequence $(x^{k},y^{k})$ weakly converges to a solution of SEP (1), which completes proof. □

## Applications

The split feasibility problem (SFP) formulated as follows:

82 $$ \mbox{Find} \quad x \in C \quad \mbox{such that} \quad Ax \in Q, $$

was originally introduced in Censor and Elfving [29]. The SFP can be a model for many inverse problems where constraints are imposed on the solutions in the domain of a linear operator as well as in the operator’s range. It has a variety of specific applications in real world, such as medical care, image reconstruction and signal processing (see [30–33] for details).

In fact, the SEP is equivalent to the SFP. Firstly, observe that the equality $Ax=By$ in (1) equals to

$$\bigl(B^{*}B\bigr)^{-1}B^{*}BAx=\bigl(B^{*}B\bigr)^{-1}\bigl(B^{*}B \bigr)y=y. $$

So, if we define the linear and bounded operator $L=(B^{*}B)^{-1}B^{*}BA:H_{1}\rightarrow H_{2}$, then the SEP becomes a special case of the SFP with the operator *L* (e.g., see [34, 35]).

On the other hand, if $H_{2}=H_{3}$ and $B=I$, then the split equality problem (1) reduces to the split feasibility problem.

Based on this equivalence, we can construct iterative algorithms for the SEP by using the algorithms for the SFP if the operator $(B^{*}B)^{-1}$ is easily computed. We also can extend the algorithms for the SEP to the SFP.

Next, we present an algorithm for the SFP based on Algorithm 3.2.

Define the function $F: H_{1} \times H_{2} \rightarrow H_{1}$ by

$$F(x,y)=A^{*}(Ax-y), $$

and the function $G: H_{1} \times H_{2} \rightarrow H_{2}$ by

$$G(x,y)=y-Ax. $$

### Algorithm 4.1

Given constants $\sigma_{0} >0$, $\alpha\in(0,1)$, $\theta\in(0,1)$ and $\rho\in(0,1)$, let $x^{0} \in H_{1}$ and $y^{0}\in H_{2}$ be taken arbitrarily.

For $k = 0,1,2,\ldots $ , compute

$$\textstyle\begin{cases} u^{k}=P_{C}(x^{k}-\beta_{k} F(x^{k},y^{k})), \\ v^{k}=P_{Q}(y^{k}-\beta_{k} G(u^{k},y^{k})), \end{cases} $$

where $\beta_{k}$ is chosen to be the largest $\beta\in\{\sigma_{k},\sigma _{k}\alpha,\sigma_{k}\alpha^{2},\ldots \}$ satisfying

$$\beta^{2}\bigl( \bigl\Vert F\bigl(x^{k},y^{k} \bigr)-F\bigl(u^{k},v^{k}\bigr) \bigr\Vert ^{2}+ \bigl\Vert y^{k}-v^{k} \bigr\Vert ^{2}\bigr)\leq \theta^{2}\bigl( \bigl\Vert x^{k}-u^{k} \bigr\Vert ^{2}+ \bigl\Vert y^{k}-v^{k} \bigr\Vert ^{2}\bigr). $$

Compute next iterates $x^{k+1}$ and $y^{k+1}$ by

$$\textstyle\begin{cases} x^{k+1}_{\mathrm{I}}=x^{k}-\gamma\rho_{k} c_{k}, \\ y^{k+1}_{\mathrm{I}}=y^{k}-\gamma\rho_{k} d_{k}, \end{cases} $$

or

$$\textstyle\begin{cases} x^{k+1}_{\mathrm{II}}=P_{C}(x^{k}-\gamma\beta _{k}\rho_{k} F(u^{k},v^{k})), \\ y^{k+1}_{\mathrm{II}}=P_{Q}(y^{k}-\gamma\beta _{k}\rho_{k} G(u^{k},v^{k})), \end{cases} $$

where $\gamma\in[0,2)$,

$$\textstyle\begin{cases} c_{k}:=(x^{k}-u^{k})-\beta_{k}(F(x^{k},y^{k})-F(u^{k},v^{k})); \\ d_{k}:=(y^{k}-v^{k})-\beta_{k}(y^{k}-v^{k}), \end{cases} $$

and

$$\rho_{k}:=\frac{\langle x^{k}-u^{k}, c_{k}\rangle+\langle y^{k}-v^{k}, d_{k}\rangle +\beta_{k}\| Au^{k}-v^{k}\|^{2}}{\|c_{k}\|^{2}+\|d_{k}\|^{2}}. $$

If

83 $$\begin{aligned}& {\beta_{k}^{2}}\bigl( \bigl\Vert F \bigl(x^{k},y^{k}\bigr)-F\bigl(x^{k+1},y^{k+1} \bigr) \bigr\Vert ^{2}+ \bigl\Vert G\bigl(x^{k},y^{k} \bigr)-G\bigl(x^{k+1},y^{k+1}\bigr) \bigr\Vert ^{2} \bigr) \\& \quad \leq\rho^{2}\bigl({ \bigl\Vert x^{k}-x^{k+1} \bigr\Vert ^{2}+ \bigl\Vert y^{k}-y^{k+1} \bigr\Vert ^{2}}\bigr), \end{aligned}$$

then set $\sigma_{k}=\sigma_{0}$; otherwise, set $\sigma_{k}=\beta_{k}$.

Using Theorem 3.3, we get the convergence of Algorithm 4.1.

### Theorem 4.1

*Let* $(x^{k},y^{k})$ *be generated by Algorithm* 4.1. *If the set of solutions of the SFP is nonempty*, *then* $(x^{k},y^{k})$ *converges weakly to a solution of SFP* (82).

### Remark 4.1

Similarly, it is easy to extend Algorithm 3.1 and Theorems 3.1 and 3.2 to the SFP. Here we omit it.

## Numerical examples

In this section, we use the numerical example in [8] to demonstrate the efficiency and advantage of Algorithms 3.1 and 3.2 by comparing them with Algorithms 1.1, 1.2 and 1.3.

We denote the vector with all elements 0 by $e_{0}$, and the vector with all elements 1 by $e_{1}$ in what follows. In the numerical results listed in the following table, ‘Iter.’ and ‘Sec.’ denote the number of iterations and the cpu time in seconds, respectively. For Algorithms 1.1, 1.2, 3.1 and 3.2, ‘InIt.’ denotes the number of total iterations of finding suitable $\beta_{k}$.

### Example 5.1

The SEP with $A=(a_{ij})_{J\times N}$, $B=(b_{ij})_{J\times M}$, $C=\{ x\in{R}^{N}\mid \|x\|\leq0.25\}$, $Q=\{ y\in{R}^{M}\mid e_{0}\leq y\leq u\} $, where $a_{ij}\in[0,1]$, $b_{ij}\in[0,1]$ and $u\in[e_{1},2e_{1}]$ are all generated uniformly randomly.

In the implementations, we take $\|Ax-By\|<\varepsilon=10^{-4}$ as the stopping criterion. Take the initial value $x_{0}=10e_{1}$, $y_{0}=-10e_{1}$.

**Figure 1:**
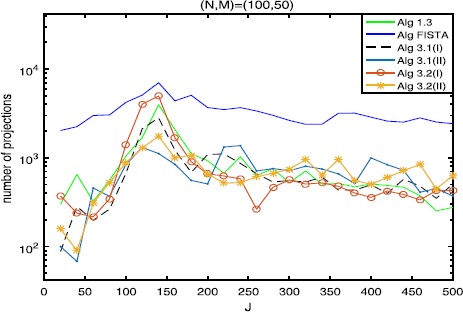
**Numbers of projections with** $\pmb{(N,M)=(100,50)}$ **.**

We make comparison of Algorithms 1.1, 1.2, 1.3, 3.1, 3.2 and FISTA with different *J*, *N*, *M*, and report the results in Tables 1, 2, 3 and Figure 1. We take the stepsize $\beta_{k}$ via a backtracking stepsize rule. For comparison, we tried to choose best parameters through numerical experiments. We take $\gamma=0.8$, $\theta=0.99$, $\sigma=50$, $\rho =0.1$ and $\alpha=0.1$ in Algorithms 1.1, 1.2, 3.1 and 3.2. And we take $\sigma_{k}=0.65$ in Algorithm 1.3. So the stepsize $\beta_{k}$ is chosen in such a way that

$$\beta_{k}=0.65 \times\min \biggl\{ \frac{\|Ax^{k}-By^{k}\|^{2}}{\|A^{*}(Ax^{k}-By^{k})\|^{2}}, \frac{\|Ax^{k}-By^{k}\|^{2}}{\|B^{*}(Ax^{k}-By^{k})\|^{2}} \biggr\} . $$

We take $L_{0}=13$, $\eta=2$ and $a=7$ for FISTA with backtracking (see [21]). For comparison, the same random values are taken in each test for different algorithms.

**Figure 2:**
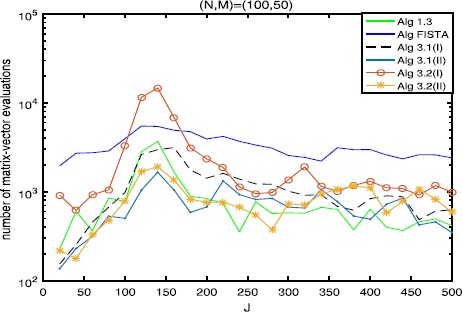
**Numbers of matrix-vector evaluations with** $\pmb{(N,M)=(100,50)}$ **.**

**Table 1:** **Computational results for Example** **5.1** **with** $\pmb{(N,M)=(100,50)}$

***J***		**50**	**100**	**150**	**200**	**250**
Algorithm 1.1	Iter.	3263	90,378	297,135	65,795	31,655
	Inlt.	13,748	377,483	864,321	172,925	82,979
	Sec.	2.188	36.672	110.563	25.500	13.781
Algorithm 1.2	Iter.	8732	194,940	434,539	82,993	43,689
	Inlt.	46,182	1,069,758	1,327,537	225,913	141,813
	Sec.	6.797	106.234	189.078	38.094	23.422
Algorithm 1.3	Iter.	336	2012	4302	1327	676
	Sec.	0.063	0.406	1.125	0.406	0.219
FISTA	Iter.	1389	2580	3787	3260	2491
	Inlt.	1397	2589	3796	3270	2501
	Sec.	0.391	0.453	0.734	0.656	0.563
Algorithm 3.1(I)	Iter.	199	793	4342	482	718
	Inlt.	254	913	5206	1145	1148
	Sec.	0.094	0.156	0.953	0.156	0.188
Algorithm 3.1(II)	Iter.	152	764	2176	580	697
	Inlt.	184	860	2232	604	769
	Sec.	0.078	0.219	0.406	0.125	0.125
Algorithm 3.2(I)	Iter.	81	2208	5704	1022	362
	Inlt.	178	4680	7562	2261	846
	Sec.	0.063	0.469	1.891	0.250	0.094
Algorithm 3.2(II)	Iter.	65	952	2311	629	263
	Inlt.	80	976	2375	653	288
	Sec.	0.031	0.188	0.516	0.156	0.063

**Table 2:** **Computational results for Example** **5.1** **with** $\pmb{(N,M)=(150,150)}$

***J***		**50**	**100**	**150**	**200**	**250**
Algorithm 1.1	Iter.	5639	14,609	36,702	895,632	364,304
	Inlt.	29,524	43,313	107,748	2,740,752	1,179,620
	Sec.	2.813	6.297	19.125	551.250	259.656
Algorithm 1.2	Iter.	9175	33,306	109,165	2,481,066	566,203
	Inlt.	28,810	139,247	389,839	10,985,694	3,467,821
	Sec.	3.906	19.922	69.797	2079.344	649.234
Algorithm 1.3	Iter.	2559	9995	40,713	1,535,172	353,573
	Sec.	0.531	3.063	16.563	793.875	219.938
FISTA	Iter.	2158	3078	3092	17,010	9264
	Inlt.	2167	3088	3102	17,021	9275
	Sec.	0.375	0.656	0.797	4.922	2.984
Algorithm 3.1(I)	Iter.	123	186	1069	26,742	8307
	Inlt.	131	690	1779	32,790	15,519
	Sec.	0.031	0.063	0.359	7.750	5.141
Algorithm 3.1(II)	Iter.	136	171	726	17,575	3765
	Inlt.	160	187	807	18,007	3813
	Sec.	0.063	0.125	0.188	5.047	1.922
Algorithm 3.2(I)	Iter.	83	808	477	27,199	10,584
	Inlt.	182	1713	1140	31,301	12,084
	Sec.	0.063	0.125	0.147	13.078	5.563
Algorithm 3.2(II)	Iter.	43	235	297	15,515	7331
	Inlt.	66	251	322	15,839	7520
	Sec.	0.006	0.063	0.094	7.094	3.750

**Table 3:** **Computational results for Example** **5.1** **with** $\pmb{(N,M)=(200,250)}$

***J***		**50**	**100**	**150**	**200**	**250**
Algorithm 1.1	Iter.	3477	6828	15,749	48,555	255,440
	Inlt.	11,943	21,084	60,677	336,456	1,326,061
	Sec.	1.688	3.844	12.672	67.453	334.891
Algorithm 1.2	Iter.	10,464	21,319	32,055	122,743	483,468
	Inlt.	30,876	86,575	162,387	420,341	2,185,371
	Sec.	5.281	16.063	32.156	111.891	600.625
Algorithm 1.3	Iter.	648	6647	16,810	44,817	132,873
	Sec.	0.188	2.781	9.500	32.734	118.203
FISTA	Iter.	2346	2931	4040	3804	6977
	Inlt.	2355	2941	4051	3815	6989
	Sec.	0.500	0.750	1.250	1.344	3.141
Algorithm 3.1(I)	Iter.	109	236	343	814	2077
	Inlt.	151	278	415	975	2518
	Sec.	0.031	0.094	0.188	0.344	7.078
Algorithm 3.1(II)	Iter.	168	188	262	756	1106
	Inlt.	180	200	268	792	1142
	Sec.	0.063	0.109	0.125	0.218	0.438
Algorithm 3.2(I)	Iter.	117	168	818	1718	1725
	Inlt.	128	222	986	2000	2408
	Sec.	0.063	0.063	0.438	1.063	1.281
Algorithm 3.2(II)	Iter.	82	83	373	582	1240
	Inlt.	98	108	400	644	1285
	Sec.	0.031	0.078	0.199	0.406	0.563

**Figure 3:**
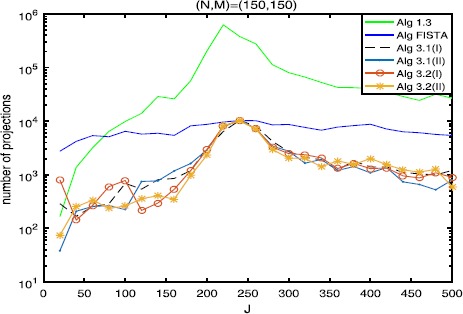
**Numbers of projections with** $\pmb{(N,M)=(150,150)}$ **.**

**Figure 4:**
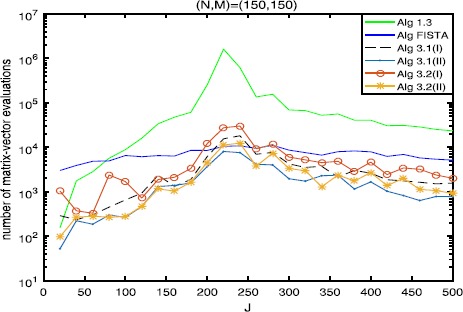
**Numbers of matrix-vector evaluations with** $\pmb{(N,M)=(150,150)}$ **.**

**Figure 5:**
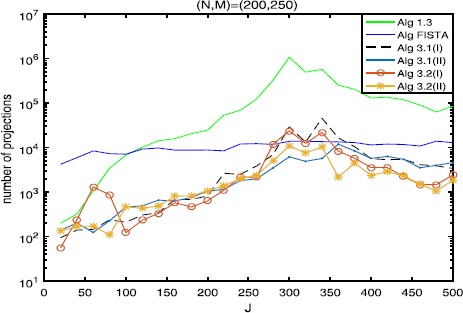
**Numbers of projections with** $\pmb{(N,M)=(200,250)}$ **.**

The numerical results are listed in Tables 1, 2, 3 and Figures 1-6, from which we can get some conclusions:

1. Algorithm 1.2 behaves worst, and Algorithm 1.1 is superior to it, while inferior to Algorithms 1.3, 3.1 and 3.2.
2. The numbers of projections and matrix-vector evaluations that Algorithms 1.3, 3.1 and 3.2 need are close when $M,N$ are small. However, the numbers of projections and matrix-vector evaluations that Algorithms 3.1 and 3.2 need are less than those of Algorithm 1.3 as *M*, *N* become bigger.
3. In Figures 1, 3 and 5, the number of projections of Algorithm 3.1(I) and (II) (or Algorithm 3.2) is close although the iteration number of Algorithm 3.1(II) is less than that of Algorithm 3.1(I). The reason is that two projections are needed in Algorithm 3.1(II) while one projection is needed in Algorithm 3.1(I) per each iteration.

**Figure 6:**
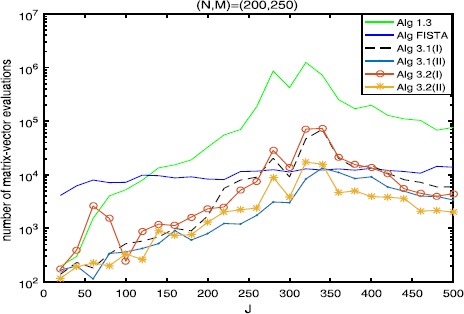
**Numbers of matrix-vector evaluations with** $\pmb{(N,M)=(200,250)}$ **.**
4. In Tables 1, 2 and 3, Algorithm 3.1(II) (or Algorithm 3.2(II)) has better performance than Algorithm 3.1(I) (or Algorithm 3.2(I)), maybe because the projections onto *C* and *Q* are very simple.
5. From Figures 1-5, it is observed that there exist peak values for Algorithms 1.3, 3.1 and 3.2, while FISTA has no peak values and is better than Algorithms 1.3, 3.1 and 3.2 near the peak values for some cases. However, for the other values of *M*, *N*, *J*, Algorithms 3.1 and 3.2 behave better than FISTA.

## Conclusion

In this article we introduce two simultaneous projection algorithms and two semi-alternating projection algorithms to solve the SEP. We present larger stepsizes in (31) and (59) than those in Algorithms 2.1 and 2.2 in [15], which leads to a better contraction property and faster convergence speed of Algorithms 3.1 and 3.2. The weak convergence for the proposed methods is proved under standard conditions.

A numerical experiment is provided to illustrate that, except for FISTA, Algorithms 1.3, 3.1 and 3.2 have peak values. It is thus natural to combine our methods with inertial effects. This is one of our future research topics.
